# Adaptation of the endemic coronaviruses HCoV-OC43 and HCoV-229E to the human host

**DOI:** 10.1093/ve/veab061

**Published:** 2021-07-22

**Authors:** Diego Forni, Rachele Cagliani, Federica Arrigoni, Martino Benvenuti, Alessandra Mozzi, Uberto Pozzoli, Mario Clerici, Luca De Gioia, Manuela Sironi

**Affiliations:** Scientific Institute IRCCS E. MEDEA, Bioinformatics, via don Luigi Monza, 23843 Bosisio Parini, Italy; Scientific Institute IRCCS E. MEDEA, Bioinformatics, via don Luigi Monza, 23843 Bosisio Parini, Italy; Department of Biotechnology and Biosciences, University of Milan-Bicocca, Piazza della Scienza, Milan 20126, Italy; Department of Biotechnology and Biosciences, University of Milan-Bicocca, Piazza della Scienza, Milan 20126, Italy; Scientific Institute IRCCS E. MEDEA, Bioinformatics, via don Luigi Monza, 23843 Bosisio Parini, Italy; Scientific Institute IRCCS E. MEDEA, Bioinformatics, via don Luigi Monza, 23843 Bosisio Parini, Italy; Department of Physiopathology and Transplantation, University of Milan, via Francesco Sforza, Milan 20122, Italy; Don C. Gnocchi Foundation ONLUS, IRCCS, Via Capecelatro, Milan 20148, Italy; Department of Biotechnology and Biosciences, University of Milan-Bicocca, Piazza della Scienza, Milan 20126, Italy; Scientific Institute IRCCS E. MEDEA, Bioinformatics, via don Luigi Monza, 23843 Bosisio Parini, Italy

**Keywords:** molecular evolution, positive selection, endemic coronavirus, molecular dating, antigenic drift, receptor binding

## Abstract

Four coronaviruses (HCoV-OC43, HCoV-HKU1, HCoV-NL63, and HCoV-229E) are endemic in human populations. All these viruses are seasonal and generate short-term immunity. Like the highly pathogenic coronaviruses, the endemic coronaviruses have zoonotic origins. Thus, understanding the evolutionary dynamics of these human viruses might provide insight into the future trajectories of SARS-CoV-2 evolution. Because the zoonotic sources of HCoV-OC43 and HCoV-229E are known, we applied a population genetics–phylogenetic approach to investigate which selective events accompanied the divergence of these viruses from the animal ones. Results indicated that positive selection drove the evolution of some accessory proteins, as well as of the membrane proteins. However, the spike proteins of both viruses and the hemagglutinin-esterase (HE) of HCoV-OC43 represented the major selection targets. Specifically, for both viruses, most positively selected sites map to the receptor-binding domains (RBDs) and are polymorphic. Molecular dating for the HCoV-229E spike protein indicated that RBD Classes I, II, III, and IV emerged 3–9 years apart. However, since the appearance of Class V (with much higher binding affinity), around 25 years ago, limited genetic diversity accumulated in the RBD. These different time intervals are not fully consistent with the hypothesis that HCoV-229E spike evolution was driven by antigenic drift. An alternative, not mutually exclusive possibility is that strains with higher affinity for the cellular receptor have out-competed strains with lower affinity. The evolution of the HCoV-OC43 spike protein was also suggested to undergo antigenic drift. However, we also found abundant signals of positive selection in HE. Whereas such signals might result from antigenic drift, as well, previous data showing co-evolution of the spike protein with HE suggest that optimization for human cell infection also drove the evolution of this virus. These data provide insight into the possible trajectories of SARS-CoV-2 evolution, especially in case the virus should become endemic.

## 1. Introduction

Coronaviruses (order *Nidovirales*, family *Coronaviridae*, subfamily *Coronavirinae*) are a diverse group of positive-sense, single-stranded RNA viruses with high zoonotic potential (Forni et al., 2017; Cui, Li, and Shi, 2019; Ye et al., 2020). Three highly pathogenic coronaviruses are now known to infect humans (SARS-CoV, MERS-CoV, and SARS-CoV-2). SARS-CoV and MERS-CoV had their zoonotic origin in palm civets and camels, respectively (Drosten et al., 2003; Zaki et al., 2012; Forni et al., 2017; Cui, Li, and Shi, 2019). Containment and surveillance strategies allowed the control of these viruses, which have never or only occasionally reappeared in human populations (Lipsitch et al., 2003). SARS-CoV-2 was first recognized in China in late 2019 and is now recognized as the cause of COVID-19 (Zhu et al., 2020). Most likely, the virus originated and evolved in bats, eventually spilling over to humans, either directly or through an intermediate host (Killerby et al., 2020; Zhou et al., 2020; Lam et al., 2020; Xiao et al., 2020; Wong et al., 2020; Liu et al., 2020; Sironi et al., 2020). To date, more than 132 million COVID-19 cases have been confirmed (https://covid19.who.int/, as of 7 April 2021), suggesting that, until an effective vaccination campaign is implemented, the virus will continue to circulate among people and, possibly, other animals (Kissler et al., 2020; Olival et al., 2020; Oude Munnink et al., 2021).

Before the emergence of these three highly pathogenic viruses, coronaviruses were considered relatively harmless to humans. In fact, four other coronaviruses (HCoV-OC43, HCoV-HKU1, HCoV-NL63, and HCoV-229E), sometimes referred to as endemic or ‘common cold coronaviruses’, have been circulating in human populations for decades, causing mild symptoms in most infected individuals (Forni et al., 2017). All these viruses are seasonal and generate short-term immunity, with reinfections being common within 1 year (Edridge et al., 2020; Galanti and Shaman, 2021).

Like the highly pathogenic coronaviruses, the endemic coronaviruses have a zoonotic origin (Forni et al., 2017; Cui, Li, and Shi, 2019). Phylogenetic analyses indicated that bats most likely represent the ultimate animal reservoirs from which the HCoV-NL63 and HCoV-229E alphacoronaviruses emerged (Corman et al., 2015; Tao et al., 2017). It is presently unknown whether HCoV-NL63 was transmitted to humans via an intermediate host, as the most closely related viruses were detected in bats from Kenya (Tao et al., 2017). Conversely, viruses highly similar to HCoV-229E were identified in camelids (dromedary camels and alpacas), strongly suggesting that, in analogy to MERS-CoV, these animals represented the zoonotic source (intermediate host) of human infection (Corman et al., 2016; Forni et al., 2017; Cui, Li, and Shi, 2019). The other two endemic coronaviruses, HCoV-OC43 and HCoV-HKU1, belong to the *Betacoronavirus* genus and most likely have their animal origin in rodents (Forni et al., 2017; Cui, Li, and Shi, 2019). Whereas it is widely accepted that bovines were the intermediate hosts mediating the transmission of HCoV-OC43 to humans, the zoonotic source of HCoV-HKU1 is presently unknown (Vijgen et al., 2005a, 2006; Forni et al., 2017; Corman et al., 2018; Cui, Li, and Shi, 2019). Given the commensal behavior of several rodents, it cannot be excluded that the virus was directly transmitted to our species by mice or related animals.

Most previous estimates indicated that the endemic coronaviruses entered human populations in the last 1,000 years (Vijgen et al., 2005a, 2006; Pfefferle et al., 2009; Huynh et al., 2012; Bidokhti et al., 2013; Al-Khannaq et al., 2016; Forni et al., 2017). However, little is known about the past and ongoing selective events that accompanied the emergence and spread of these viruses in human populations. Recent works (Jo, Drosten, and Drexler, 2021; Kistler and Bedford, 2021) focused on the spike proteins of the endemic coronaviruses and, by analyzing extant genetic diversity, detected evidence of positive selection in the receptor-binding domain (RBD). Here, we exploited the availability of animal viruses closely related to HCoV-229E and HCoV-OC43 to apply a method that jointly analyzes inter- and intra-specific diversity. Analysis of all coding sequences indicated that positive selection is not limited to the spike protein, and phylogenetic inference suggested that antigenic drift is not the only explanation for the selection signals in the RBD.

## 2. Materials and methods

### 2.1 Sequence selection and recombination analysis

Complete or almost complete genome sequences of HCoV-229E (*n* = 31) and HCoV-OC43 coronaviruses (*n* = 165) were downloaded from the National Center for Biotechnology Information (NCBI) database (http://www.ncbi.nlm.nih.gov/, last accessed 7 April 2021). Only sequences with known sampling dates were included in the analyses (Supplementary Table S1). The HCoV-OC43 Paris strain was excluded as its sampling date is uncertain (Vijgen et al., 2005b). For both human coronaviruses, the reference sequences of the closest phylogenetically related animal virus were also retrieved: camel alphacoronavirus (NC_028752) for HCoV-229E and BCoV (NC_003045) for HCoV-OC43. These sequences were used as outgroups in gammaMap analysis (see paragraph 2.2).

All complete genome sequences with sampling year of BCoV were also retrieved from NCBI (*n* = 92) (Supplementary Table S2 and Supplementary Table S1).

The alignment of all viral open reading frames (ORFs) was analyzed for evidence of recombination. In particular, we applied five methods implemented in RDP4 (RDP, GENECONV, MaxChi, Chimera, and 3Seq) (Sawyer, 1989; Smith, 1992; Martin and Rybicki, 2000; Posada and Crandall, 2001; Martin et al., 2017; Lam, Ratmann, and Boni, 2018). Recombination events with a *P* value <0.01 for at least three methods were considered as significant. Recombinant sequences were removed from downstream analyses.

### 2.2 Population genetics–phylogenetic analysis

Selective events that accompanied the appearance of the human viruses were investigated for HCoV-OC43 and HCoV-229E—the two endemic coronaviruses for which the closest related animal virus is likely known (i.e. the bovine and the camelid coronaviruses).

Analyses were performed with gammaMap (Wilson et al., 2011), which uses intra-species variation and inter-species diversity to estimate the distribution of selection coefficients (γ). A Bayesian sliding window approach along coding sequences is used to infer changes in the selective pressure and estimate the posterior probability of γ for each codon (Wilson et al., 2011). Thus, all ORF sequences of the two coronaviruses were retrieved and all possible overlapping regions were masked.

Single ORF alignments were generated using MAFFT (v7.3) (Katoh and Standley, 2013) implemented in the RevTrans 2.0 utility (Wernersson and Pedersen, 2003). This tool takes the information of the protein sequence alignment as a scaffold for constructing the corresponding codon multiple alignment. Gaps occur in groups of three and cover an entire codon, therefore codon boundaries are maintained.

GammaMap categorizes selection coefficients into twelve predefined classes ranging from −500 (inviable) to 100 (strongly beneficial), with zero indicating neutrality (Wilson et al., 2011). We also assumed that θ (neutral mutation rate per site), *k* (transitions/transversions ratio), and *T* (branch length) vary along genes following lognormal distributions, whereas *P* (probability of adjacent codons sharing the same selection coefficient) following a log-uniform distribution. Finally, for the selection coefficients, we considered a uniform Dirichlet distribution with the same prior weight for each selection class. We performed two runs with 100,000 iterations each and with a thinning interval of ten iterations. Runs were merged after checking for convergence. Codon positions were defined as positively selected if they showed a posterior probability > 0.75 of having γ ≥ 1.

Sequence logos were generated using WebLogo (Crooks et al., 2004) (https://weblogo.berkeley.edu/, last accessed 28 May 2021).

### 2.3 Molecular modeling and epitope prediction

The structure of HCoV-229E RBD of Class I in complex with human aminopeptidase N (hANPEP) was retrieved from the Protein Data Bank (PDB ID: 6ATK). Such structure was also used as template to model the interaction between the S protein RBD of HCoV-229E camel ortholog and camel ANPEP (cANPEP), using the webserver HOMCOS (Kawabata, 2016). The HOMCOS webserver performs blastp searches (Altschul et al., 1997) to look for complexes formed by proteins, which are homologous to the query proteins. We then selected one of these complexes as a template and launched the program MODELLER (Šali and Blundell, 1993) that models the interaction between the query proteins with a script provided by HOMCOS. On the basis of sequence similarity at the binding interface, we chose structures 6U7E, 6U7F, and 6U7G as templates to model the interaction of RBD Class I–II, Class IV, and Class V/VI, respectively, with hANPEP. The same templates have been used to model the sole RBDs using SWISS-MODEL (Arnold et al., 2006). These RBD structures were then used to map epitopes on the molecular surface. Volume, Area, Dihedral Angle Reporter (VADAR) 1.8 (Willard et al., 2003) was used to assess the accuracy of all models. VADAR uses a combination of more than fifteen specific algorithms to calculate different parameters for each residue and for the overall protein structure. We used such parameters to verify (1) the agreement of observed structural parameters (such as φ and ψ dihedral and buried charges) of the newly predicted structures with the expected values calculated on the corresponding sequences and (2) the presence of a low number of packing defects. Structures were then analyzed using the software PyMOL (Schrödinger, 2017) that was also used to create protein figures.

The structures of HCoV-OC43 HE and BCoV HE proteins have also been retrieved from PDB (PDB IDs: 5N11 and 3CL5, respectively). Missing loops in the 3CL5 structure (such as β5–β6) have been modeled using MODELLER. Epitope positions were predicted using the BepiPred-2.0 method with default parameters and accessed through the Immune Epitope Database (IEDB) server (http://tools.iedb.org/bcell/help/#Bepipred-2.0) (Jespersen et al., 2017, last accessed 28 May 2021).

### 2.4 Temporal signal and molecular dating

Molecular dating of the HCoV-229E RBD was performed using a set of ninety-five sequences (Supplementary Table S1). These included the ones deriving from complete genomes (*N* = 30, with the exclusion of one recombinant) plus sixty-five partial genomes. These were selected from public repositories because they have complete sequence information for the RBD and a known collection date. These sequences do not necessarily represent complete spike proteins. Recombination analysis was performed on this extended data set using RDP4, as reported above. No significant evidence of recombination was detected.

To evaluate whether the HCoV-229E RBD spike region carried sufficient temporal signal, we calculated the correlation coefficients (*r*) of regressions of root-to-tip genetic distances against sequence sampling years (Murray et al., 2016). We applied a method that minimizes the residual mean squares of the models and calculated *P* values by performing clustered permutations (1,000) of the sampling dates (Duchene et al., 2015; Murray et al., 2016). This method is robust to situations where the temporal and the genetic structures are confounded (i.e. where closely related sequences were preferentially sampled at the same time) (Duchene et al., 2015; Murray et al., 2016). HCoV-229E RBD spike proteins showed evidence of temporal signal (Supplementary Fig. S1).

To infer the best nucleotide substitution model, we run the JmodelTest software (Posada and Crandall, 1998). Results indicated the generalized time-reversible model as preferred, with gamma distributed rate variation among sites and proportion of invariable sites. The stepping-stone sampling method (Xie et al., 2011) implemented in the BEAST package was applied to select the best-fit molecular clock and tree prior. We evaluated a strict and a lognormal relaxed clock with a constant size, an exponential growth, or a coalescent Bayesian skyline tree prior and we compared their corresponding marginal likelihoods. For each of the six models, we run 100 steps, 1,000,000 iterations each. A model is considered to be favored if the Bayes factor (BF) is more than two. The stepping-stone sampling showed that the model with a BF > 2 compared to each of all other models was the lognormal relaxed clock with a coalescent constant tree prior.

Two final analyses were run for 50 million generations each, with 10 per cent burn-in, and sampled every 5,000 steps. The two runs were combined after checking for convergence with the Tracer tool (Rambaut et al., 2018) and for having effective sampling sizes >100 for all parameters. A maximum clade credibility tree using TreeAnnotator (Bouckaert et al., 2014) was generated and visualized with FigTree (http://tree.bio.ed.ac.uk/, last accessed 28 May 2021).

## 3. Results

### 3.1 Recent and ongoing evolution of HCoV-OC43 and HCoV-229E

Coronaviruses have large and complex genomes which encode sixteen non-structural (nsps) and four structural proteins (spike, envelope, membrane, and nucleoprotein), as well as a variable number of accessory molecules. Embecoviruses (e.g. HCoV-OC43, HCoV-HKU1, and BCoV) encode an additional structural protein, a hemagglutinin-esterase (HE), which serves as a receptor-destroying enzyme (de Groot, 2006; Forni et al., 2017). Analysis of bat viruses indicated that SARS-CoV-2 required limited adaptation to gain the ability to infect our species and to spread via human-to-human transmission (Cagliani et al., 2020; MacLean et al., 2020). In analogy to SARS-CoV-2, human endemic coronaviruses have zoonotic origins, and understanding their emergence as human pathogens might provide insight into possible future dynamics of SARS-CoV-2 evolution. We thus focused on HCoV-OC43 and HCoV-229E, as their zoonotic sources are relatively certain, to investigate the selective patterns acting on their coding regions since the separation from bovine/camel viruses. To this aim, we first screened viral ORF alignments for the presence of recombination events (see Methods), which were detected in the spike proteins of both viruses. Recombinant sequences (one for HCoV-229E and thirty three for HCoV-OC43) were removed from the data set. We next applied gammaMap (Wilson et al., 2011), a method that combines analysis of within-population variation and divergence from an outgroup to estimate codon-wise selection coefficients (γ).

**Figure 1.**
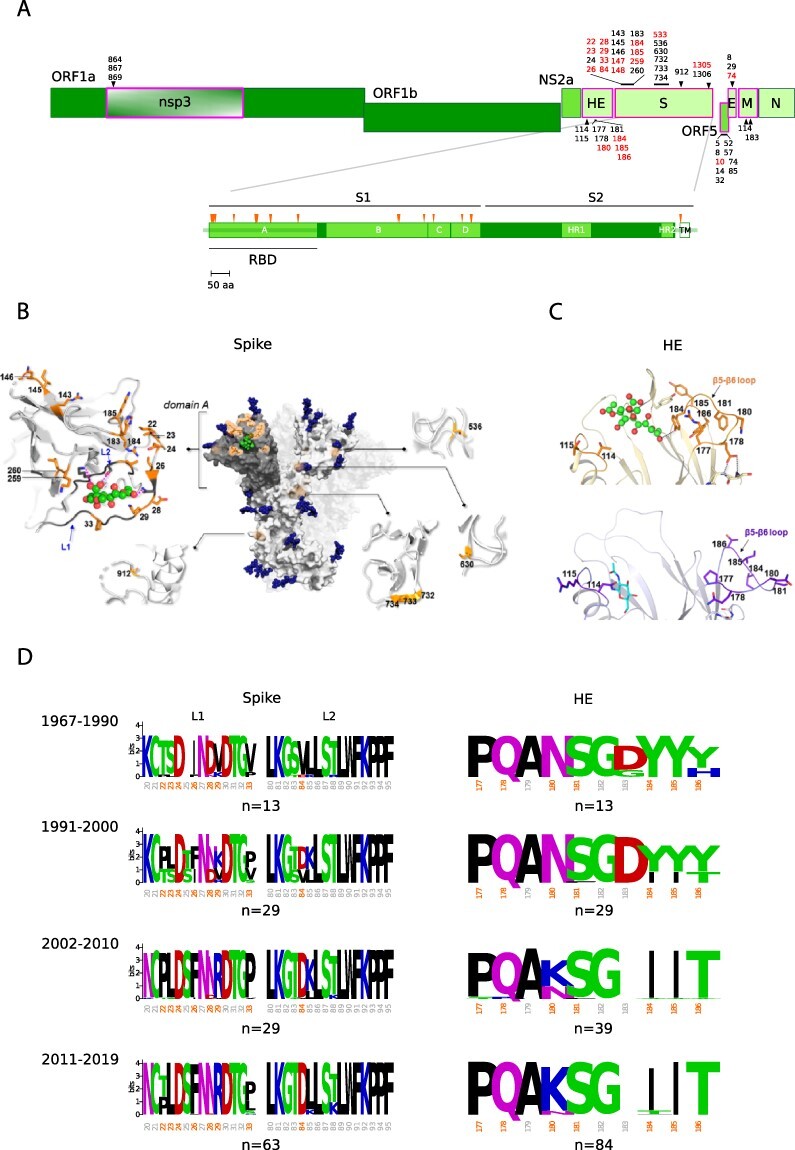
Positive selection acting on HCoV-OC43. (A) A schematic representation of HCoV-OC43 ORFs is reported with indication of all positively selected sites found by gammaMap. Sites that have a frequency of the most common amino acid <0.95 are shown in red. ORFs having sites with evidence of positive selection are boxed in magenta. A schematic representation of the spike protein is also reported (Walls et al., 2016). (B) Positively selected sites (orange) mapped on the 3D structure of HCoV-OC43 spike protein trimer (PDB: 6NZK) in complex with a 9-*O*-acetylated sialic acid. For clarity, only the positively selected sites on one monomer of the spike protein are shown. Secondary structure features are depicted as gray cartoons, while side chains of positively selected sites are represented as sticks: C atoms are colored in orange, N atoms in blue, and O atoms in red. Glycans are rendered as dark blue spheres except for the 9-*O*-acetylated sialic acid with its C atoms in green, O atoms in red, and N atoms in blue. Structural details of portions of the spike protein where positively selected sites were identified are also reported. In particular, positively selected sites at Domain A, together with the 9-*O*-acetylated sialic acid binding site are shown. Loop 1 and Loop 2, defining the pocket, are also indicated (L1 and L2, blue labels). (C) Ribbon representation of a portion of HCoV-OC43 (light orange, PDB ID: 5N11) and BCoV HE proteins (light purple, PDB ID: 3CL5). Positively selected sites are shown with side chains been explicated as orange and purple sticks, respectively. Color codes: carbon, orange (HCoV-OC43) or purple (BCoV); oxygen, red; nitrogen, blue. Hydrogens have been omitted for clarity. (D) Sequence logos of the RBD Loop 1 and Loop 2 of the spike protein and 177–196 region of the HE protein. Positively selected sites are shown in orange (irrespective of their amino acid frequency). Missing information implies a gap. Sequence logos are grouped by collection date. Numbering refers to the HCoV-OC43 reference sequence (NC_006213).

**Figure 2.**
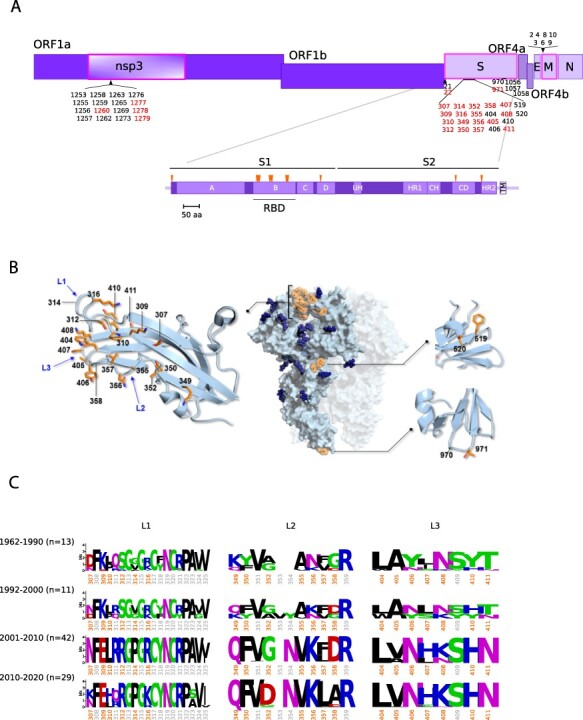
Positive selection acting on HCoV-229E. (A) A schematic representation of HCoV-229E ORFs is reported with indication of all positively selected sites found by gammaMap. Sites with a frequency of the most common amino acid <0.95 are shown in red. ORFs having sites with evidence of positive selection are boxed in magenta. A schematic representation of the spike protein is also reported (Li et al., 2019). (B) Positively selected sites mapped on the 3D structure of HCoV-E229 spike protein (PDB: 6U7H). For clarity, only the positively selected sites (in orange) on one monomer of the spike protein are shown. Structural details of portions of the spike protein are also reported, in particular three RBD loops (L1, L2, and L3, blue labels). Secondary structure features are depicted as light blue cartoons, while side chains of positively selected sites are represented as sticks: C atoms are colored in orange, N atoms in blue, and O atoms in red. Glycans are rendered as dark blue spheres. (C) Sequence logos of the three RBD loops. Positively selected sites are shown in orange (irrespective of their amino acid frequency). Sequence logos are grouped by collection date. Numbering refers to the HCoV-229E reference sequence (NC_002645).

In line with data on several other viruses (Ho et al., 2011; Cagliani et al., 2020), we found that most codons evolved under strong to moderate purifying selection (γ < −5) (Supplementary Fig. S2). However, sites with robust evidence of positive selection (posterior probability > 0.75 of γ ≥ 1) could also be detected. The majority of these sites are located in a restricted number of proteins with mainly structural functions (Figs. 1A and 2A, and Supplementary Fig. S3). In particular, for HCoV-229E and HCoV-OC43, 64.4 per cent and 52.7 per cent of positively selected sites are located in the spike protein. Whereas most selected sites in the spike proteins and in HE are polymorphic in circulating viral populations (suggesting ongoing selection), those located in other regions are not (Supplementary Table S3). In line with previous findings (Jo, Drosten, and Drexler, 2021; Kistler and Bedford, 2021), the positively selected sites in the spike proteins of both HCoV-OC43 and HCoV-229E are clustered within regions that interact with the cellular receptors (9-*O*-acetylated sialoglycans, Sia-9-O and aminopeptidase N, ANPEP) and that were previously shown to modulate binding (Figs. 1 and 2). Thus, several positively selected sites are located within the sialoglycan-binding site of the HCoV-OC43 spike protein. Specifically, Sites 29 and 259 are within the binding pocket (Fig. 1B), whereas changes at Sites 22 and 24 in other embecoviruses largely affect binding affinity (Hulswit et al., 2019). Similarly, the HE positively selected sites map to the lectin domain. Mutations at several positively selected sites determine the loss of sialoglycan binding, which is thought to have contributed to the shift to the human host (Bakkers et al., 2017). Specifically, the N114 change (T114 in BCoV) creates a glycosylation site that greatly decreases sugar binding (Bakkers et al., 2017). The Y184I substitution results in the loss of a hydrogen bond with the Sia-9-O ligand, whereas changes at Sites 177 and 178 affect the conformation of the loop β5-β6, eventually decreasing binding (Bakkers et al., 2017). Most likely, the same applies to changes at Positions 185 and 186 (Fig. 1C). With respect to HCoV-229E, most positively selected sites in the S protein map to the three loops that contact human ANPEP (hANPEP) (see paragraph 3.2) (Wong et al., 2017; Li et al., 2019). Overall, these data indicate that gammaMap reliably identified relevant selection signatures.

We next analyzed polymorphic positively selected sites in the S and HE proteins by grouping viruses collected in 10-year intervals (with the first interval spanning a longer period to include early samples). Although the number of sequences in each interval differs, the amount of observed polymorphisms does not seem to be related to the sample size. Overall, the binning into time intervals suggests that the evolution of HCoV-OC43 and HCoV-229E is ongoing and that new amino acid combinations have progressively emerged (Figs. 1D and 2C). Indeed, the amino acid status at the positively selected sites broadly corresponds to the RBD classes of HCoV-229E and to HCoV-OC43 genotypes. Interestingly, analysis of the RBD region in ninety-two BCoV sequences revealed limited variability with no clear temporal pattern (Supplementary Fig. S4). The same comparison could not be performed for camelid viruses as most of them were sampled in 2014–2015.

### 3.2 Evolution of HCoV-229E optimized receptor binding

**Figure 3.**
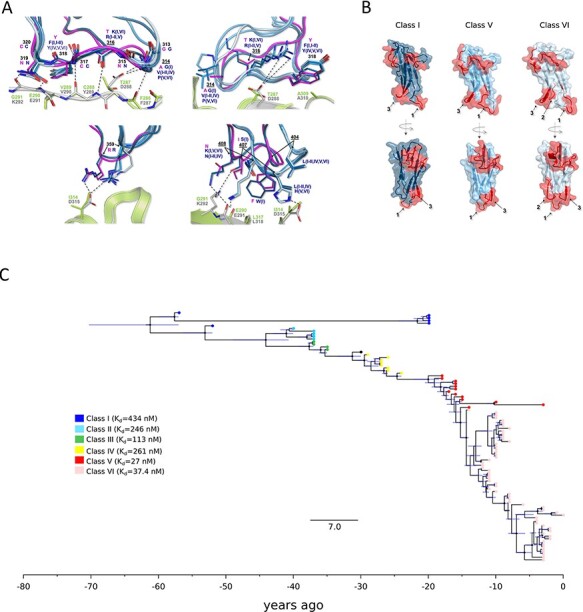
Molecular evolution of HCoV-229E spike protein. (A) Atomic details of the interactions between HCoV-229E (six RBD classes, blue shades) and the corresponding model for camel alphacoronavirus (pink) with both hANPEP (gray) and cANPEP (light green). RBDs-hANPEP conserved interaction pattern, involving Loop 1, is shown in the upper-left panel. Relevant structural differences among the various RBDs and the details on their interactions at the binding interface are represented in the other three panels. Positively selected sites are underlined. N atoms are colored in blue and O atoms are in red. Salt bridges and H-bonds are represented by dashed black lines. (B) Epitopes mapped on the spike protein RBDs of three different HCoV-E229 classes. The RBDs are in different shades of blue, whereas the epitopes are in red. Labels 1, 2, and 3 refer to Loop 1, Loop 2, and Loop 3. (C) Timescaled maximum clade credibility tree of the spike protein RBD. Branch lengths represent the evolutionary time measured by the grids corresponding to the timescale shown at the tree base (in years). For internal nodes, 95 per cent credible interval bars are shown and black dots indicate a posterior probability >0.80 for that node. Tip nodes are colored based on the figure legend, where the *K*_d_ of all six RBD classes for hANPEP interaction calculated by Wong et al., (2017) is also reported.

Because, in analogy to SARS-CoV-2, HCoV-229E binds a protein receptor, we further investigated the positively selected sites in the spike protein. The specificity of HCoV-229E for hANPEP was previously ascribed to an extended tandem of H-bonds involving the 314–320 segment of RBD Loop 1 and the 287–292 portion of a surface-exposed β-strand hANPEP Domain II (Wong et al., 2017). Most of these interactions involve backbone atoms, reducing the dependency on sequence variations. In fact, the camel alphacoronavirus can use hANPEP as a receptor (Corman et al., 2016). It was, however, suggested that changes in loop regions might accommodate species-specific differences among ANPEP orthologs and optimize receptor-binding affinity (Li et al., 2019). We thus compared the HCoV-229E RBD crystal structure and the corresponding model for camel alphacoronavirus (Fig. 3A). We also modeled camel ANPEP (cANPEP) based on the structure of the human ortholog. Overall, cANPEP features fewer charged residues at the interface than the human protein. In particular, T287 and I314 are replaced by D288 and D315 in the human receptor, whereas G291 is replaced by K292. Analysis of the contact interface indicated that the Positively Selected Sites 316 (R or K, depending on RBD class), 407 (S in Class I and H in Classes V and VI), and 408 (K in Classes I, V, and VI) contribute to additional interactions with the human protein than those established by the camel virus (Fig. 3A). These are made possible by the presence of the charged residues in the human receptor. Overall, these observations suggest that HCoV-229E can interact with hANPEP more efficiently than the camel virus and that positively selected sites contribute to increased affinity.

Previous investigations showed that the affinity of the six RBD classes of HCoV-229E for hANPEP varies in a range of K_d_ from ∼430 nM (Class I) to ∼30 nM (Classes V and VI) (Fig. 3C) (Wong et al., 2017). In particular, a strong increase in affinity is observed for Classes V and VI. Some of the positively selected sites contribute to this increased affinity by changing loop conformation and by establishing additional interactions (Supplementary Fig. S5). For instance, H407 in Classes V and VI forms an additional polar interaction with the spatially close D315 of hANPEP, and K408 in the same classes intercepts the E291 backbone in the receptor (Fig. 3A).

Variations in the RBD loops, which progressively emerged over the last 50 years (Fig. 3C), were previously proposed to derive from immune selection (Wong et al., 2017; Jo, Drosten, and Drexler, 2021; Kistler and Bedford, 2021). Inspection of the IEDB database revealed that no experimental epitope for the spike protein of HCoV-229E has been described. We thus used the sequences of RBDs belonging to different classes to predict epitope positions using BepiPred-2. Results indicated that epitopes do differ among RBD classes (Supplementary Table S4) and map to different structural regions (Fig. 3B) (data for HCoV-OC43 are shown in Supplementary Fig. S6). This is in line with the observation that antibodies against Classes I and IV show no cross-neutralization and that HCoV-229E is undergoing antigenic drift (Wong et al., 2017; Li et al., 2019; Eguia et al., 2020; Eguia et al., 2021). Nonetheless, the hypothesis that antigenic drift is the only driver of S protein evolution is difficult to reconcile with the evidence that reinfection with HCoV-229E is common and humoral immunity is short-lived (Edridge et al., 2020; Galanti and Shaman, 2021).

To clarify these issues, we used an extended set of spike protein sequences (*n* = 95) to date the temporal emergence of RBD classes. The spike protein data set had a robust temporal signal (Supplementary Fig. S1), allowing application of molecular dating approaches. Results indicated that Classes II, III, and IV, which have about twofold higher affinity than Class I, emerged 3–9 years apart (Fig. 3C). However, since the appearance of Class V (with much higher affinity) about 25 years ago, no RBD class emerged for 10 years (Fig. 3C). In fact, Class VI split from Class V about 15 years ago and the two classes show very similar sequence and binding properties. Thus, little variation seems to have accumulated approximately in the last 25 years. These different time intervals are not fully consistent with antigenic drift, which is expected to result in a more regular emergence of antigenic variants. An alternative, not mutually exclusive possibility is that strains with higher affinity for the cellular receptor have out-competed strains with lower affinity and that HCoV-229E has evolved to optimize binding to the cellular receptor.

## 4. Discussion

Zoonotic diseases have been constantly emerging during human history, accounting for a large number of epidemics and pandemics, as well as for an enormous health burden. The endemic coronaviruses usually cause very mild symptoms, at least in immunocompetent individuals, and can hardly be regarded as pathogens of concern. We however mention that, because they have now circulated in (and adapted to) human populations for decades or centuries, it cannot be excluded that they were once more pathogenic than they are now. Although we cannot go back in time and infer the original phenotype of endemic coronaviruses, nor can we have a full picture of their ancestral genetic diversity, analysis of their evolution is potentially very informative to understand the future trajectories of SARS-CoV-2 and of coronaviruses in general. Analysis of bat coronaviruses indicated that, in analogy to SARS-CoV, SARS-CoV-2 required limited adaptation to gain the ability to infect and spread in our species (Cagliani et al., 2020; MacLean et al., 2020). As HCoV-OC43 and HCoV-229E most likely emerged from bovine and camelid coronaviruses, we investigated which selective events accompanied the divergence of these human viruses from the animal ones and their diffusion in humans. We note, however, that because of the lack of information on early isolates, it is formally impossible to distinguish between the initial events associated with the optimization for human infection and the ongoing adaptation resulting from immune selection or other pressures.

Our results indicate that the spike protein and other structural proteins of both viruses represented the major targets of selection. An interesting exception is the strong signature of selection we observed for HCoV-OC43 ORF5 (also known as ns12.9). The encoded protein functions as a viroporin and its deletion reduces viral replication, inflammatory response, and virulence in mouse models (Zhang et al., 2015). Positive selection also drove the evolution of the membrane proteins of both viruses, as well as of the envelope protein of HCoV-OC43. This latter, besides having structural roles, acts as a viroporin and represents a neurovirulence factor (Stodola et al., 2018). Likewise, the membrane proteins of several coronaviruses, including HCoV-OC43, in addition to their role in virion maturation, are capable of antagonizing interferon responses (Yang et al., 2013; Siu et al., 2014; Beidas and Chehadeh, 2018). Overall, these data suggest that positively selected sites in these proteins might contribute to fine-tuning the interaction between coronaviruses and human immune responses.

Clearly, the spike protein and HE in the case of HCoV-OC43 have a major interest as targets of selection, as they represent major determinants of host range and infectivity (Forni et al., 2017; Cui, Li, and Shi, 2019). Most selected sites were found to be located in the RBDs of the spike proteins, as well as in the lectin domain of HE. However, additional sites mapped to other regions of the spike proteins and were mostly fixed in frequency. These include three sites in the heptad repeat region of the spike protein of HCoV-229E and one site in the fusion peptide of HCoV-OC43 (Figs 1. and 2). Notably, the heptad repeat region was previously described as a major target of selection in MERS-CoV and related camel viruses (Cotten et al., 2014; Forni et al., 2015) and variants within this region and/or the fusion peptide were shown to modulate viral tropism and host range in several viruses, including animal coronaviruses (de Haan et al., 2006; Yamada et al., 2009). It is also worth mentioning that, in line with our data, a previous analysis that focused on the spike proteins detected positive selection for both HCoV-OC43 and HCoV-229E, although the sites did not exactly correspond to the ones we describe herein (Jo, Drosten, and Drexler, 2021). The reason for this is that different methodologies were applied to search for selection signatures. Specifically, we used a method that jointly uses divergence (from the outgroup) and genetic diversity (within the sampled human viruses) to detect selection events that occurred since the separation from the bovine or camel viruses. As a consequence, the selected sites detected by gammaMap can be either fixed or polymorphic in circulating human strains. Conversely, Jo and coworkers did not include outgroup information and used methods that detected sites with dN/dS significantly higher than one in the sampled population of human viruses (Jo, Drosten, and Drexler, 2021).

Coronaviruses can use very different cellular receptors and their spike proteins display a remarkable ability to adapt to different cellular receptors (Forni et al., 2017). Embecoviruses such as HCoV-OC43, HCoV-HKU1, and BCoV attach to 9-*O*-acetylated sialoglycans via the spike protein, with HE acting as a receptor-destroying enzyme (de Groot, 2006; Hulswit et al., 2019). Conversely, HCoV-229E and HCoV-NL63 use a protein receptor (Forni et al., 2017). Biochemical and crystallographic analyses indicated that, since the shift to the human host, the spike and HE proteins of HCoV-OC43 have co-evolved to optimize the balance between binding and release from sialoglycans in human airways (Bakkers et al., 2017; Lang et al., 2020). We confirm herein the previously observed emergence of spike and HE variants over time and the replacement of earlier variants with the more recent ones (Fig. 1). However, the relative binding affinity of HE and spike variants have not been extensively investigated, yet. This fact, the complex interplay between the two proteins and the poor knowledge of the structure of 9-*O*-acetylated sialoglycoconjugates that are effectively bound in the human respiratory tract make it impossible to analyze in detail affinity changes over time. Conversely, binding assays have shown that different classes of the HCoV-229E spike protein RBD have very different binding affinities for hANPEP. The appearance of variants with increased affinity has clearly occurred progressively in time (Fig. 3C), as a result of positive selection (Fig. 3A). On one hand, these data suggest that HCoV-OC43 and HCoV-229E have been adapting to optimize receptor engagement and spread in human populations. On the other hand, the evolution of the spike proteins of endemic coronaviruses has been interpreted in terms of antigenic drift (Wong et al., 2017; Li et al., 2019; Eguia et al., 2020; Eguia et al., 2021; Jo, Drosten, and Drexler, 2021; Kistler and Bedford, 2021). Indeed, it was previously demonstrated that antibodies raised against HCoV-229E Class I RBD do not neutralize viruses with RBDs belonging to different classes (Wong et al., 2017; Li et al., 2019). Along the same lines, Eguia and coworkers showed that human sera collected in the 1980s and 1990s have low neutralizing activity against the spike proteins from HCoV-229E strains isolated years later (Eguia et al., 2020; Eguia et al., 2021). This is a clear indication that HCoV-229E has undergone antigenic drift. However, these observations do not imply that immune escape is the only driver of HCoV-229E evolution. In fact, growing evidence suggests that the humoral immune response against endemic coronaviruses wanes in a few months (Kiyuka et al., 2018; Edridge et al., 2020; Galanti and Shaman, 2021). As a consequence, natural reinfection is common between 6 and 105 months (Edridge et al., 2020). Thus, it is unclear whether the antibody response can be regarded as a strong selective pressure for these viruses. Our dating of the emergence of HCoV-229E RBD classes indicates an initial rapid turnover of Classes I–IV followed by a 10-year time during which no variant turned up after the emergence of Class V. Class V and the closely related Class VI RBDs differ in binding affinity from the other classes by almost an order of magnitude (Wong et al., 2017). Since the emergence of these high-affinity classes, the HCoV-229E spike proteins have accumulated fewer changes compared to earlier time periods. These patterns are not readily explained by the antigenic drift hypothesis, which predicts a more regular emergence of spike variants. Thus, together with the remarkable seasonality of endemic coronaviruses, these patterns suggest that selection has also been acting to optimize binding to the cellular receptor and that strains with increasing affinity have replaced those with lower binding ability and, possibly, lower infectivity. In this respect, it is also worth mentioning that even Class V and VI RBDs have much lower affinity for hANPEP (K_d_ ∼30 nM) than most other human coronaviruses for their respective cellular receptors (K_d_ in the range of 1–5 nM for SARS-CoV-2, SARS-CoV, and HCoV-NL63) (Wu et al., 2009; Yi et al., 2020). It is thus possible that optimization for receptor binding played a relevant role for HCoV-229E evolution.

We also found signals of positive selection in the lectin domain of HE. Whereas such signals might also result from antigenic drift (Jo, Drosten, and Drexler, 2021; Kistler and Bedford, 2021), previous data showing co-evolution of the spike protein with HE (Bakkers et al., 2017; Lang et al., 2020) suggest that optimization for human cell infection contributed to the evolution of this virus. In this respect, it is interesting to note that very limited variation with no temporal pattern was evident in the RBD region of BCoV sequences sampled over 34 years. This suggests that, if antigenic drive occurs in humans, it does not in cattle.

The observations above are not meant to imply that immune escape played no role in the evolution of HCoV-229E and HCoV-OC43 and, most likely, distinct coronaviruses are subject to diverse selective pressures. For instance, Kistler and Bedford detected no evidence of antigenic drift for HCoV-NL63 (Kistler and Bedford, 2021). Clearly, gaining insight into the evolution of the other human coronaviruses has relevance for our understanding of SARS-CoV-2. Recent work has indicated that the spike protein of SARS-CoV-2 can tolerate a substantial number of substitutions, with some of them even increasing receptor binding (Starr et al., 2020). The N501Y substitution in the RBD is one such variant and it is shared by three of the recently emerged SARS-CoV-2 lineages (B.1.1.7, P.1, and B.1.351), which also carry a number of additional replacements in the spike (https://www.gov.uk/government/publications/investigation-of-novel-sars-cov-2-variant-variant-of-concern-20201201, last accessed 28 May 2021) (Faria et al., 2021; Tegally et al., 2021). The initial characterization of these lineages has indicated that B.1.1.7 is more transmissible than previous lineages (Leung et al., 2021), but seems to have similar antigenic properties as the prototypic strain (Xie et al., 2021; Muik et al., 2021; Wang et al., 2021; Hoffmann et al., 2021). Conversely, B.1.351 and P.1, both carrying the E484K substitution in the RBD, have been associated with cases of reinfection (Resende et al., 2020; Kuzmina et al., 2021; Nonaka et al., 2021; Naveca et al., 2021) and evasion of naturally elicited or vaccine-elicited antibody responses (Wang et al., 2021; Hoffmann et al., 2021; Zhou et al., 2021; Jangra et al., 2021; Li et al., 2021). Albeit very preliminary, these observations suggest that SARS-CoV-2 can adapt to elude previous immunity. Notably, the mass deployment of vaccines against SARS-CoV-2 will subject the virus to a selective pressure that the endemic coronaviruses have never experienced.

## Supplementary Material

veab061_Supp

## Data Availability

Sequences were retrieved from the NCBI (http://www.ncbi.nlm.nih.gov/, last accessed 7 April 2021) database. Lists of all accession IDs are reported in Supplementary Table S1 and Supplementary Table S2.
