# Supplementary material for: Adaptation of the endemic coronaviruses HCoV-OC43 and HCoV-229E to the human host
Source: Virus Evol. 2021 Jun 24;7(2):veab061. doi: 10.1093/ve/veab061 (PMC8344746; doi:10.1093/ve/veab061)

Supplementary Material

Adaptation of the endemic coronaviruses HCoV-OC43 and HCoV-229E to the human host

Supplementary Table 1. List of Human coronavirus OC43 and Human coronavirus 229E coronavirus strains.

HCoV-OC43

Accession ID	Species	Collection Date
NC_006213	Human coronavirus OC43	1967
KY014281	Human coronavirus OC43	2002
AY903459[+]	Human coronavirus OC43	2004
AY903460	Human coronavirus OC43	2004
KY014282[+]	Human coronavirus OC43	2007
KY983583	Human coronavirus OC43	2015
KY983585	Human coronavirus OC43	2015
KY983588	Human coronavirus OC43	2015
KY967356	Human coronavirus OC43	2015
KY967358	Human coronavirus OC43	2015
KY967359	Human coronavirus OC43	2015
KY967360[+]	Human coronavirus OC43	2015
KY967361	Human coronavirus OC43	2015
KY554972	Human coronavirus OC43	2016
KY554973	Human coronavirus OC43	2016
KY554974	Human coronavirus OC43	2016
KY554975	Human coronavirus OC43	2016
KY674917	Human coronavirus OC43	2016
KY674918	Human coronavirus OC43	2016
KY674920	Human coronavirus OC43	2016
KY684759	Human coronavirus OC43	2016
KY369905	Human coronavirus OC43	2016
KY369906	Human coronavirus OC43	2016
KY369907	Human coronavirus OC43	2016
MN306036[+]	Human coronavirus OC43	2019
MN306041[+]	Human coronavirus OC43	2019
MN306042[+]	Human coronavirus OC43	2019
MN306043[+]	Human coronavirus OC43	2019
MN306053[+]	Human coronavirus OC43	2019
MN310476[+]	Human coronavirus OC43	2019
MN310478[+]	Human coronavirus OC43	2019
KF530093	Human coronavirus OC43	1983
KF530060	Human coronavirus OC43	1985
KF530085	Human coronavirus OC43	1987
KF530086	Human coronavirus OC43	1987
KF530087	Human coronavirus OC43	1987
KF530077	Human coronavirus OC43	1987
KF530083	Human coronavirus OC43	1987
KF530073	Human coronavirus OC43	1989
KF530066	Human coronavirus OC43	1990
KF530065	Human coronavirus OC43	1990

KF530061	Human coronavirus OC43	1990
KF530088	Human coronavirus OC43	1990
KF530076	Human coronavirus OC43	1991
KF530096	Human coronavirus OC43	1991
KF530091	Human coronavirus OC43	1991
KF530089	Human coronavirus OC43	1991
KF530095	Human coronavirus OC43	1991
KF530067	Human coronavirus OC43	1991
KF530082	Human coronavirus OC43	1991
KF530094	Human coronavirus OC43	1991
KF530079	Human coronavirus OC43	1991
KF530071	Human coronavirus OC43	1992
KF530097	Human coronavirus OC43	1992
KF530074	Human coronavirus OC43	1992
KF530090	Human coronavirus OC43	1993
KF530059	Human coronavirus OC43	1995
KF530084	Human coronavirus OC43	1995
KF530062	Human coronavirus OC43	1995
KF530075	Human coronavirus OC43	1995
KF530098	Human coronavirus OC43	1996
KF530064	Human coronavirus OC43	1996
KF530078	Human coronavirus OC43	1996
KF530063	Human coronavirus OC43	1996
KF530099	Human coronavirus OC43	1997
KF530072	Human coronavirus OC43	1997
KF530080	Human coronavirus OC43	1997
KF530069	Human coronavirus OC43	1998
KF530081	Human coronavirus OC43	1999
KF530070	Human coronavirus OC43	1999
KF530068	Human coronavirus OC43	2000
KF530092	Human coronavirus OC43	2000
KF923905	Human coronavirus OC43	2005
KF923889[+]	Human coronavirus OC43	2006
KF923899	Human coronavirus OC43	2006
KF923900	Human coronavirus OC43	2006
KF923890	Human coronavirus OC43	2007
KF923892	Human coronavirus OC43	2007
KF923894	Human coronavirus OC43	2007
KF923907	Human coronavirus OC43	2007
KF923921	Human coronavirus OC43	2007
KF923891	Human coronavirus OC43	2007
KF923901	Human coronavirus OC43	2007
KF923908[+]	Human coronavirus OC43	2007
KF923909	Human coronavirus OC43	2007
KF923910	Human coronavirus OC43	2007
KF923911	Human coronavirus OC43	2007
KF923912	Human coronavirus OC43	2007
KF923913	Human coronavirus OC43	2007
KF923914	Human coronavirus OC43	2007

KF923915	Human coronavirus OC43	2007
KF923916	Human coronavirus OC43	2007
KF923917	Human coronavirus OC43	2007
KF923919	Human coronavirus OC43	2007
KF923920	Human coronavirus OC43	2007
KF923923	Human coronavirus OC43	2008
KF923922	Human coronavirus OC43	2009
KP198611[+]	Human coronavirus OC43	2010
KF923886[+]	Human coronavirus OC43	2010
KF923887[+]	Human coronavirus OC43	2010
KF923918	Human coronavirus OC43	2010
KP198610[+]	Human coronavirus OC43	2010
KF923893	Human coronavirus OC43	2010
KF923895[+]	Human coronavirus OC43	2010
KF923924	Human coronavirus OC43	2010
KF923888[+]	Human coronavirus OC43	2010
KF923925	Human coronavirus OC43	2010
KX344031	Human coronavirus OC43	2011
KU131570[+]	Human coronavirus OC43	2011
KJ958218	Human coronavirus OC43	2011
KJ958219	Human coronavirus OC43	2011
KF923896[+]	Human coronavirus OC43	2012
KX538964	Human coronavirus OC43	2012
KF923898[+]	Human coronavirus OC43	2012
KF923906[+]	Human coronavirus OC43	2012
KX538965	Human coronavirus OC43	2012
KX538966	Human coronavirus OC43	2012
KF923902	Human coronavirus OC43	2012
KF923903	Human coronavirus OC43	2012
KF923904	Human coronavirus OC43	2012
KX538967	Human coronavirus OC43	2012
KX538968	Human coronavirus OC43	2012
KX538969	Human coronavirus OC43	2012
KF923897	Human coronavirus OC43	2012
KX538970	Human coronavirus OC43	2012
KX538971	Human coronavirus OC43	2012
KX538972	Human coronavirus OC43	2012
KX538973	Human coronavirus OC43	2012
KX538974	Human coronavirus OC43	2012
KX538975	Human coronavirus OC43	2012
KX538976	Human coronavirus OC43	2012
KX538977	Human coronavirus OC43	2012
KX538978	Human coronavirus OC43	2013
KX538979	Human coronavirus OC43	2013
MG197718[+]	Human coronavirus OC43	2015
MG197709[+]	Human coronavirus OC43	2015
MG197710	Human coronavirus OC43	2015
MG197715	Human coronavirus OC43	2015
MG197719[+]	Human coronavirus OC43	2015

MG197720[+]	Human coronavirus OC43	2015
MG197716	Human coronavirus OC43	2015
MG197711[+]	Human coronavirus OC43	2015
MG197712	Human coronavirus OC43	2015
MG197721[+]	Human coronavirus OC43	2015
MG197717	Human coronavirus OC43	2015
MG197714[+]	Human coronavirus OC43	2015
MG197713	Human coronavirus OC43	2015
MG197722	Human coronavirus OC43	2015
MG977446	Human coronavirus OC43	2016
MF374983	Human coronavirus OC43	2016
MF314143[+]	Human coronavirus OC43	2016
MG197723	Human coronavirus OC43	2016
MG977451	Human coronavirus OC43	2016
MG977452	Human coronavirus OC43	2016
MH121121	Human coronavirus OC43	2016
MG977447	Human coronavirus OC43	2016
MG977448	Human coronavirus OC43	2016
MG977449	Human coronavirus OC43	2016
MG977450	Human coronavirus OC43	2016
MG977445	Human coronavirus OC43	2016
MF374984	Human coronavirus OC43	2017
MG977444	Human coronavirus OC43	2017
MF374985	Human coronavirus OC43	2017
MN026165[+]	Human coronavirus OC43	2017
MN026164	Human coronavirus OC43	2018
NC_003045	Bovine coronavirus (Betacoronavirus 2)	

HCoV-229E

Accession ID	Species	Collection Date
NC_002645	Human coronavirus 229E	1962
JX503061	Human coronavirus 229E	2009
JX503060	Human coronavirus 229E	2010
KY983587	Human coronavirus 229E	2015
KY967357	Human coronavirus 229E	2015
KU291448	Human coronavirus 229E	2015
KY621348	Human coronavirus 229E	2016
KY674914	Human coronavirus 229E	2016
KY674919	Human coronavirus 229E	2016
KY684760	Human coronavirus 229E	2016
KY369908	Human coronavirus 229E	2016
KY369909	Human coronavirus 229E	2016
KY369910	Human coronavirus 229E	2016
KY369911	Human coronavirus 229E	2016
KY369912	Human coronavirus 229E	2016
KY369913	Human coronavirus 229E	2016

KY369914	Human coronavirus 229E	2016
MT438696	Human coronavirus 229E	2016
MF542265	Human coronavirus 229E	2016
MN369046	Human coronavirus 229E	2018
MN306046	Human coronavirus 229E	2019
KF514429	Human coronavirus 229E	1989
KF514432	Human coronavirus 229E	1993
KF514433	Human coronavirus 229E	1993
KF514430	Human coronavirus 229E	1993
KF514431	Human coronavirus 229E	1995
MT438700	Human coronavirus 229E	2017
MT438697	Human coronavirus 229E	2017
MT438698	Human coronavirus 229E	2017
MT438699	Human coronavirus 229E	2017
MW039392[+]	Human coronavirus 229E	2020
AY386390[*]	Human coronavirus 229E	1967
AB691764[*]	Human coronavirus 229E	2004
DQ243964[*]	Human coronavirus 229E	1979
DQ243965[*]	Human coronavirus 229E	1982
DQ243966[*]	Human coronavirus 229E	1982
DQ243967[*]	Human coronavirus 229E	1982
DQ243968[*]	Human coronavirus 229E	1982
DQ243969[*]	Human coronavirus 229E	1982
DQ243970[*]	Human coronavirus 229E	1982
DQ243971[*]	Human coronavirus 229E	1984
DQ243972[*]	Human coronavirus 229E	1984
DQ243973[*]	Human coronavirus 229E	1990
DQ243974[*]	Human coronavirus 229E	1992
DQ243975[*]	Human coronavirus 229E	1992
DQ243976[*]	Human coronavirus 229E	1992
AY386388[*]	Human coronavirus 229E	1999
AY386389[*]	Human coronavirus 229E	1999
AY386386[*]	Human coronavirus 229E	1999
AY386383[*]	Human coronavirus 229E	1999
DQ243977[*]	Human coronavirus 229E	2001
DQ243978[*]	Human coronavirus 229E	2001
DQ243979[*]	Human coronavirus 229E	2002
DQ243980[*]	Human coronavirus 229E	2003
DQ243983[*]	Human coronavirus 229E	2003
DQ243984[*]	Human coronavirus 229E	2003
DQ243985[*]	Human coronavirus 229E	2003
DQ243986[*]	Human coronavirus 229E	2003
AB691765[*]	Human coronavirus 229E	2004
KM055557[*]	Human coronavirus 229E	2005
KM055559[*]	Human coronavirus 229E	2005
KM055551[*]	Human coronavirus 229E	2007
KM055552[*]	Human coronavirus 229E	2007
GU068546[*]	Human coronavirus 229E	2007
AB691767[*]	Human coronavirus 229E	2008

KM055554*	Human coronavirus 229E	2008
KM055555*	Human coronavirus 229E	2008
KM055556*	Human coronavirus 229E	2008
GU068547*	Human coronavirus 229E	2008
GU068548*	Human coronavirus 229E	2008
GU068549*	Human coronavirus 229E	2008
KM055532*	Human coronavirus 229E	2009
KM055533*	Human coronavirus 229E	2009
KM055534*	Human coronavirus 229E	2009
KM055535*	Human coronavirus 229E	2009
KM055536*	Human coronavirus 229E	2009
KM055537*	Human coronavirus 229E	2009
KM055545*	Human coronavirus 229E	2009
KM055546*	Human coronavirus 229E	2009
KM055547*	Human coronavirus 229E	2009
KM055553*	Human coronavirus 229E	2009
KM055558*	Human coronavirus 229E	2009
KM055560*	Human coronavirus 229E	2009
KM055538*	Human coronavirus 229E	2010
KM055539*	Human coronavirus 229E	2010
KM055540*	Human coronavirus 229E	2010
KM055541*	Human coronavirus 229E	2010
KM055542*	Human coronavirus 229E	2010
KM055543*	Human coronavirus 229E	2010
KM055531*	Human coronavirus 229E	2011
KM055544*	Human coronavirus 229E	2011
KM055548*	Human coronavirus 229E	2011
KM055549*	Human coronavirus 229E	2011
KM055550*	Human coronavirus 229E	2011
JX513245*	Human coronavirus 229E	2011
KJ866102*	Human coronavirus 229E	2013
NC_028752	Camel alphacoronavirus	2015

* strain used only in the spike RBD analyses

+ evidence of recombination in the *S* gene

Supplementary Table 2: List of BCoV strains.

Accession ID	Species	Collection Date
NC_003045.1	Bovine coronavirus	1997
DQ811784.2	Bovine coronavirus	1983
FJ425184.1	Waterbuck coronavirus	1994
FJ425185.1	Waterbuck coronavirus	1994
FJ425186.1	Waterbuck coronavirus	1994
FJ425187.1	White-tailed deer coronavirus	1994
FJ425188.1	Sambar deer coronavirus	1994
FJ425189.1	Sambar deer coronavirus	1994
FJ425190.1	Sambar deer coronavirus	1994
FJ938066.1	Bovine respiratory coronavirus	1996
FJ938063.1	Bovine coronavirus	1996
FJ938064.1	Bovine coronavirus	2000
FJ938065.1	Bovine respiratory coronavirus	2000
LC494164.1	Bovine coronavirus	2006
LC494162.1	Bovine coronavirus	2006
LC494163.1	Bovine coronavirus	2006
LC494160.1	Bovine coronavirus	2007
LC494161.1	Bovine coronavirus	2007
LC494172.1	Bovine coronavirus	2007
LC494173.1	Bovine coronavirus	2008
LC494174.1	Bovine coronavirus	2008
LC494177.1	Bovine coronavirus	2008
LC494165.1	Bovine coronavirus	2008
LC494166.1	Bovine coronavirus	2008
LC494167.1	Bovine coronavirus	2008
LC494168.1	Bovine coronavirus	2009
LC494169.1	Bovine coronavirus	2009
LC494170.1	Bovine coronavirus	2010
LC494171.1	Bovine coronavirus	2010
LC494129.1	Bovine coronavirus	2010

LC494130.1	Bovine coronavirus	2010
LC494131.1	Bovine coronavirus	2011
LC494132.1	Bovine coronavirus	2011
LC494133.1	Bovine coronavirus	2011
LC494135.1	Bovine coronavirus	2012
LC494136.1	Bovine coronavirus	2012
LC494127.1	Bovine coronavirus	2012
LC494128.1	Bovine coronavirus	2012
LC494134.1	Bovine coronavirus	2012
LC494137.1	Bovine coronavirus	2013
MG757138.1	Bovine coronavirus	2014
MG757139.1	Bovine coronavirus	2014
MG757140.1	Bovine coronavirus	2014
MG757141.1	Bovine coronavirus	2014
MG757142.1	Bovine coronavirus	2014
KX982264.1	Bovine coronavirus	2014
LC494154.1	Bovine coronavirus	2014
LC494155.1	Bovine coronavirus	2014
LC494156.1	Bovine coronavirus	2014
LC494138.1	Bovine coronavirus	2015
KU886219.1	Bovine coronavirus	2015
LC494139.1	Bovine coronavirus	2015
LC494157.1	Bovine coronavirus	2015
LC494140.1	Bovine coronavirus	2015
LC494175.1	Bovine coronavirus	2016
LC494176.1	Bovine coronavirus	2016
LC494126.1	Bovine coronavirus	2016
LC494141.1	Bovine coronavirus	2016
LC494158.1	Bovine coronavirus	2016
LC494146.1	Bovine coronavirus	2016
MH043955.1	Bovine coronavirus	2016
LC494147.1	Bovine coronavirus	2016
LC494142.1	Bovine coronavirus	2016
LC494143.1	Bovine coronavirus	2016

LC494144.1	Bovine coronavirus	2016
LC494145.1	Bovine coronavirus	2016
LC494148.1	Bovine coronavirus	2016
LC494149.1	Bovine coronavirus	2016
LC494178.1	Bovine coronavirus	2016
LC494179.1	Bovine coronavirus	2016
LC494180.1	Bovine coronavirus	2016
LC494181.1	Bovine coronavirus	2016
LC494182.1	Bovine coronavirus	2016
LC494150.1	Bovine coronavirus	2017
LC494151.1	Bovine coronavirus	2017
LC494152.1	Bovine coronavirus	2017
LC494153.1	Bovine coronavirus	2017
MG518518.1	Water deer coronavirus	2017
MH043952.1	Bovine coronavirus	2017
MH043953.1	Bovine coronavirus	2017
MH043954.1	Bovine coronavirus	2017
LC494159.1	Bovine coronavirus	2017
LC494183.1	Bovine coronavirus	2017
LC494184.1	Bovine coronavirus	2017
LC494185.1	Bovine coronavirus	2017
LC494186.1	Bovine coronavirus	2017
LC494187.1	Bovine coronavirus	2017
LC494188.1	Bovine coronavirus	2017
LC494189.1	Bovine coronavirus	2017
LC494190.1	Bovine coronavirus	2017
LC494191.1	Bovine coronavirus	2017
LC494192.1	Bovine coronavirus	2017

Supplementary Table 3. HCoV-229E and HCoV-OC43 positively selected sites.

HCoV-229E					
**Protein**	**aa position**	**Ancestral allele***	**Major allele**	**Major allele frequency**	**Posterior probability**
**ORF1a**	1253	Gly	Ile	1	0.94
	1255	Pro	Asn	1	1.00
	1256	Pro	Thr	1	1.00
	1257	Ala	Val	1	1.00
	1258	Glu	Asp	1	1.00
	1259	Leu	Thr	1	1.00
	1260	Ala	Ile	0.89	1.00
	1262	Ile	Lys	1	1.00
	1263	Asp	Glu	1	0.99
	1265	Thr	Phe	1	0.99
	1269	Val	Glu	1	0.98
	1273	Ser	Ala	1	0.97
	1276	Thr	Val	1	0.97
	1277	Cys	His	0.89	0.97
	1278	Asn	Gly	0.89	0.97
	1279	Ser	Val	0.69	0.93
**Spike**	21	Val	Gly	1	0.8
	22	Leu	Thr	0.73	0.79
	307	Asp	Lys	0.53	0.81
	309	Lys	Glu	0.8	0.85
	310	Pro	His	0.7	0.87
	312	Ser	Arg	0.8	0.85
	314	Ala	Pro	0.8	0.82
	316	Thr	Lys	0.73	0.75
	349	Lys	Gln	0.97	0.82
	350	Tyr	Phe	0.97	0.89
	352	Ala	Asp	0.7	0.98
	355	Ser	Val	0.83	0.99
	356	Asn	Lys	0.97	0.99
	357	Val	Leu	0.73	0.99
	358	Gly	Ala	0.67	0.98
	404	Phe	Leu	0.97	0.91
	405	Ala	Val	0.73	0.94
	406	Tyr	Asn	0.97	0.97
	407	Ile	His	0.63	0.98
	408	Asn	Lys	0.77	0.95
	410	Tyr	His	0.97	0.92
	411	Thr	Asn	0.9	0.9
	519	Ser	Phe	1	0.83
	520	Ser	Thr	1	0.79
	970	Asn	Gly	1	0.89
	971	Thr	Ile	0.63	0.87

	1056	Gly	Val	1	0.76
	1057	Ile	Val	1	0.76
	1058	Asp	Glu	1	0.75
**M**	2	Thr	Ser	1	0.97
	3	Glu	Asn	1	0.98
	4	Ala	Asp	1	0.98
	6	Ile	Cys	1	0.98
	8	Glu	Gly	1	0.97
	9	Glu	Asp	1	0.96
	10	Leu	Ile	1	0.95

HCoV-OC43

Protein	aa position	Ancestral allele*	Major allele	Major allele frequency	Posterior probability
**ORF1a**	864	Asn	Lys	1	0.76
	867	Ala	Ile	1	0.82
	869	Thr	Met	0.95	0.8
**E**	8	Phe	Leu	1	0.94
	29	Ile	Thr	1	0.93
	74	Val	Ile	0.75	0.8
**HE**	114	Thr	Asn	1	0.98
	115	Thr	Arg	0.99	0.99
	177	Arg	Pro	0.99	0.84
	178	Glu	Gln	0.99	0.86
	180	Asn	Lys	0.63	0.86
	181	Phe	Ser	0.99	0.87
	184	Tyr	Ile	0.75	0.92
	185	Tyr	Ile	0.8	0.91
	186	Tyr	Thr	0.8	0.9
**Spike**	22	Thr	Pro	0.57	0.82
	23	Thr	Leu	0.82	0.99
	24	Val	Asp	1	1
	26	Ile	Phe	0.82	0.99
	28	Asp	Asn	0.79	0.98
	29	Val	Arg	0.7	0.99
	33	Val	Pro	0.6	0.84
	84	Leu	Asp	0.82	0.91
	143	His	Arg	1	0.9
	145	Thr	Ile	1	0.94
	146	Ile	Asn	1	0.96
	147	Leu	Ser	0.81	0.96
	148	Gly	Thr	0.82	0.94
	183	Gln	His	1	0.88
	184	Arg	Phe	0.82	0.99

	185	Val	Lys	0.83	0.99
	259	Ala	Arg	0.82	0.99
	260	Leu	Phe	0.99	0.97
	533	Ala	Phe	0.68	0.79
	536	Thr	Pro	0.99	0.89
	630	Thr	Ser	0.99	0.78
	732	Ser	Ala	0.99	0.9
	733	Ser	Ile	1	0.95
	734	Val	Ser	1	0.96
	912	Ser	Asp	1	0.95
	1305	Gly	Cys	0.75	0.79
	1306	Phe	Leu	1	0.79
**ORF5**	5	Lys	Arg	1	0.95
	8	Ile	Lys	1	0.99
	10	Tyr	Phe	0.74	0.84
	14	Thr	Ile	0.99	0.92
	32	Lys	Gln	1	0.9
	52	Ser	Arg	0.95	0.92
	57	Leu	Phe	1	0.91
	74	Thr	Asn	1	0.91
	85	Glu	Asp	1	0.9
**M**	114	Trp	Phe	1	0.96
	183	Leu	Cys	1	0.88

* Ancestral allele refers to the outgroup sequence (Camel alphacoronavirus NC_028752 for HCoV-299E and Bovine coronavirus NC_003045 for HCoV-OC43)

Supplementary Table 4. List of predicted linear B cell epitopes for the spike proteins of HCoV-229E and HCoV-OC43.

HCoV-229E		
Isolate genBank ID (Class)	Sequence	Length
NC_002645 (Class I)	KPQSGGGKC	9
	CPFSFGKVNNFVKF	14
	LKDIPGG	7
	WAYSKY	6
DQ243968 (Class I/II)	KLQSGVGR	8
	GNCPFSFGKVNNFVKFG	17
	LKDIPGG	7
DQ243976 (class IV)	RSGVGRCYNC	10
	NCPFSFGKVNNFVK	14
	KDIPGG	6
	LANLNSH	7
KM055554 (class V)	RGPGRCYNCRP	11
	FNETKG	6
	NCPFSFGKVNNFVKF	15
	LKDIPGG	7
	LVNHKSH	7
KY369908 (Class VI)	FEHQRGPGK	9
	NVKLAR	6
	NCPFSFGKVNNFVKF	15
	LKDIPGG	7
	LVNHKS	6

HCoV-OC43		
Isolate genBank ID	Sequence	Length
NC_006213	TSDNINDKDTGPPPIST	17
	PTSGSTYRNMALK	13
	FKPPFL	6
	KVIKDRVMY	9
	TINSTQDGDNKL	12

	EYPQTICHPNLGNHRKELWHLDTGV	25
	MSDFMSEI	8
	MSDFMSEI	8
KF530093	TSDTSYINDVDTGVPPIST	19
	PTSGSTYRNMAL	12
	KVIKDDVL	8
	TINLDNKL	8
	EYPHTICHPNLGNHRTELWHLDTGV	25
	KRKDGFT	7
	MSDFMSEI	8
KF530071	LKCPLDTSYKGTFNNKDTGPPFIST	25
	PTSGSTYRNMALKGTDK	17
	TLWFKPPFL	9
	NTKVFKDGVMY	11
	TINSQDGDNKL	11
	EYPHTICHPKLGNHFKELWHLDTGV	25
	RRDIGFT	7
	MSDFMSEI	8
KF923918	NCPLDPRLKGSFNNRDTGPPSIST	24
	PTSGSTYRNMALKGTDL	17
	WFKPPFL	7
	NTKVFKDGVMY	11
	TINSTQDGVNKL	12
	EYPHTICHPKLGNHFKELWHSDTGV	25
	RLDIGFT	7
KY983583	CTLDPRLKGSFNNRDTGPPSISI	23
	PTSGSTYRNMALK	13
	FKPPFL	6
	TKVFKDGVMY	10
	TINSTQDGVNKL	12
	YPHTICHPNLGNHFKELWHYDTGV	24
	CMSDFM	6

Supplementary Figure 1. Regression plot of the root-to-tip distance against sampling dates for the RBD domain of the HCoV-229E spike protein. Each point corresponds to a viral sequence (n=95) and the line is the linear regression calculate using a method that minimizes the residual mean squares. Thirty seven sequences have overlapping values. The r coefficient and the *p* value calculated on 1000 permutations are shown. A phylogenetic tree with the generated clusters based on tip distributions is also reported.

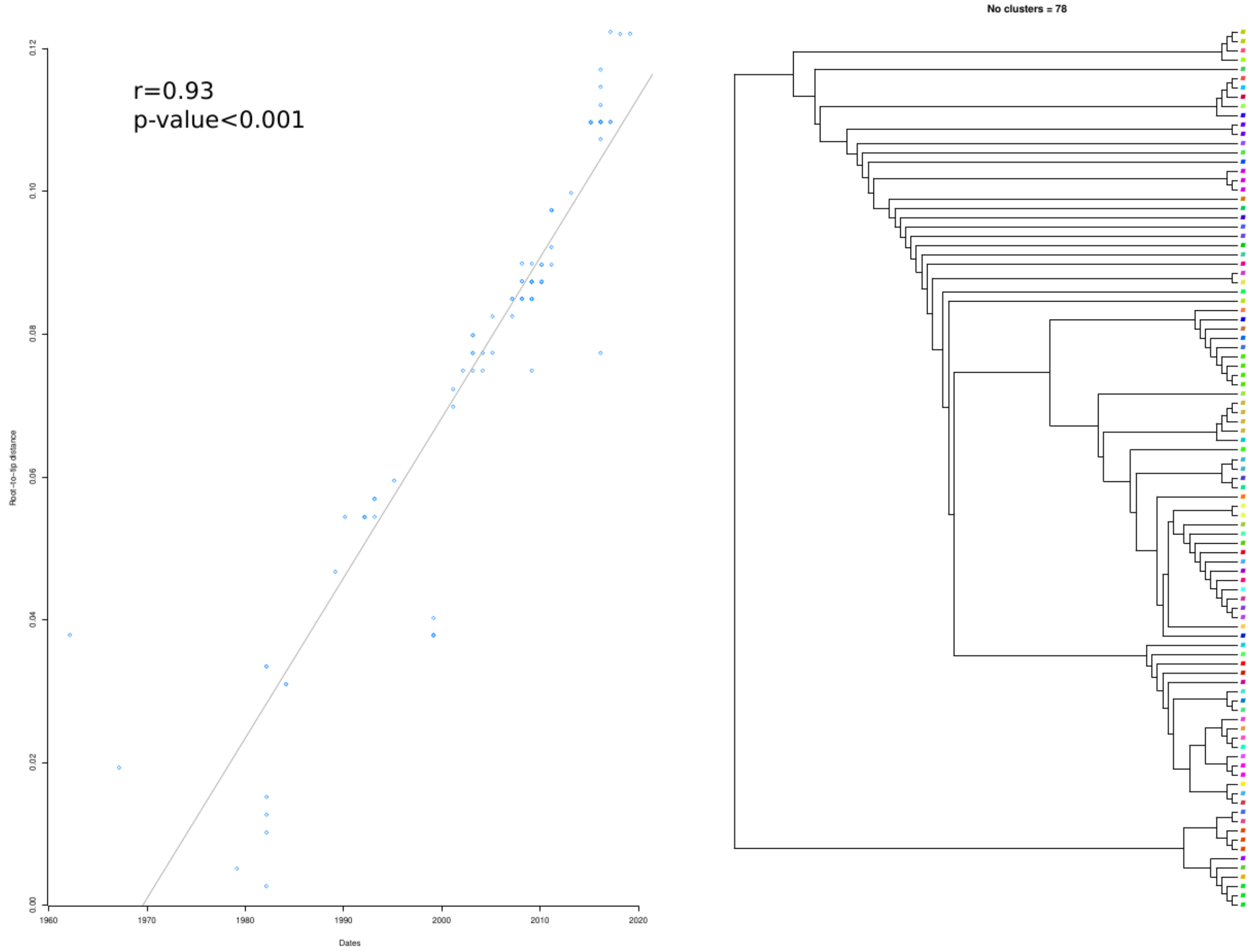

Supplementary Figure 2. Population genetics-phylogenetics analysis of HCoV-OC43 and HCoV-229E genes. Violin plots (median, white dot; interquartile range, black bar) of selection coefficients calculated for all HCoV-OC43 and HCoV-229E ORFs. Selection coefficients (γ) are classified as strongly beneficial (100, 50), moderately beneficial (10, 5), weakly beneficial (1), neutral (0), weakly deleterious (−1), moderately deleterious (−5, −10), strongly deleterious (−50, −100), and inviable (−500).

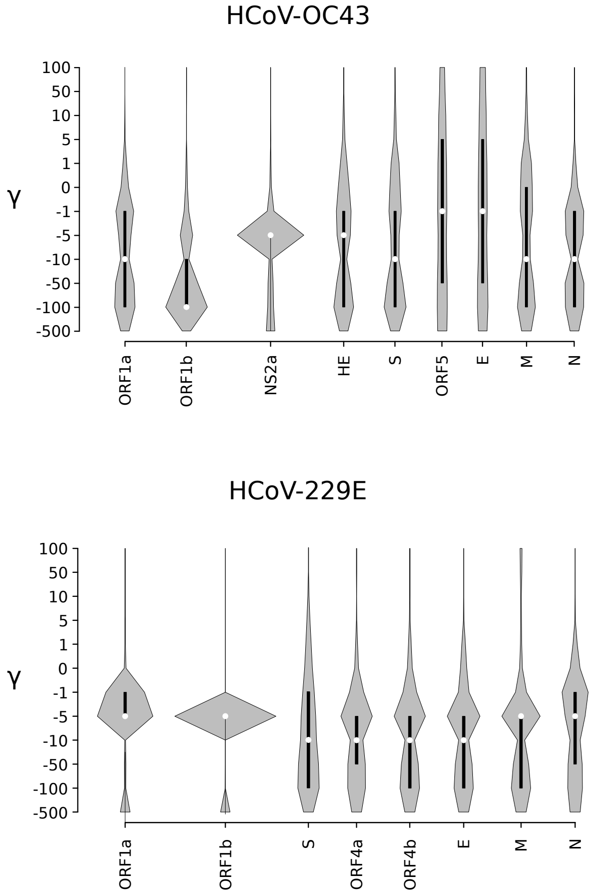

Supplementary Figure 3. Schematic representation of HCoV-OC43 and HCoV-229E proteins showing evidence of positive selection. Positions of positively selected sites detected by gammaMap are indicated with orange triangles.

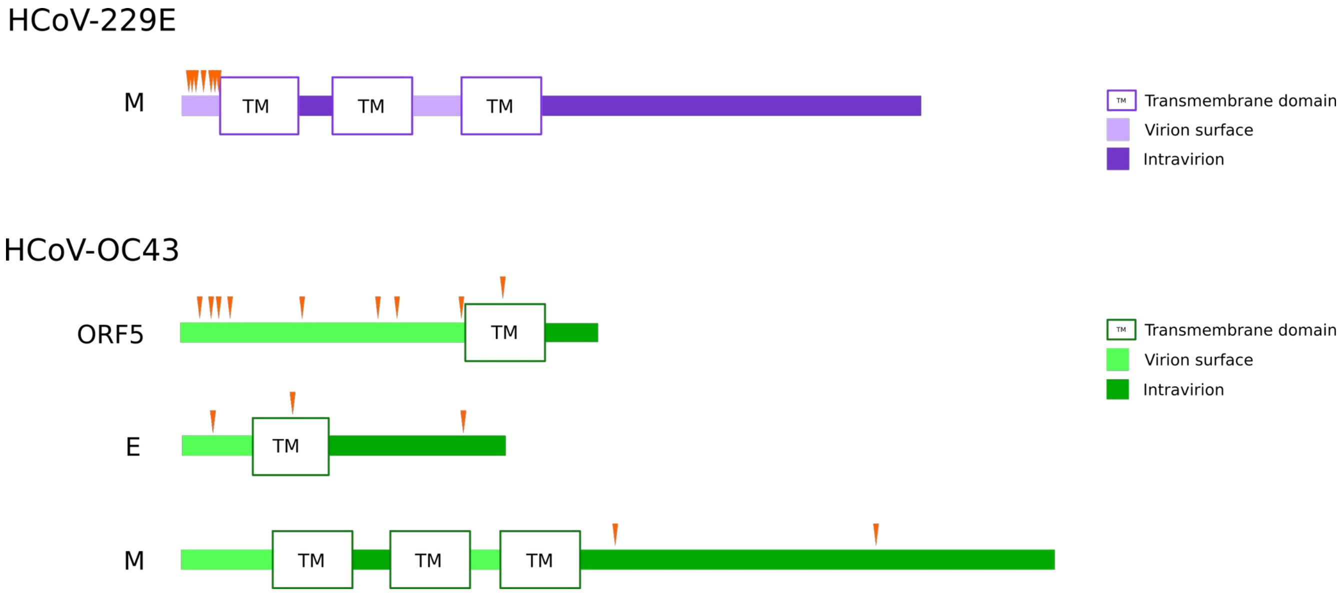

Supplementary Figure 4. Protein alignment of the three RBD loops in the spike protein of BCoV. Positions refer to NC_003045.

```
                    20                36 78                       98 162                               194
DQ811784_1983     KCTTVSINDVDTGVPSI...MALKGTLLLSTLWFKPPFLSD...YTMCEYPNTICHPTLGNQRVELWHWDTGVVSCL
FJ425184_1994     KCTTVSINDVDTGVPSI...MALKGTLLLSTLWFKPPFLSD...YTMCEYPNTICNPNLGNQRVELWHWDTGVVSCL
FJ425185_1994     KCTTVSINDVDTGVPSI...MALKGTLLLSTLWFKPPFLSD...YTMCEYPNTICNPNLGNQRVELWHWDTGVVSCL
FJ425186_1994     KCTTVSINDVDTGVPSI...MALKGTLLLSTLWFKPPFLSD...YTMCEYPNTICNPNLGNQRVELWHWDTGVVSCL
FJ425187_1994     KCTTVSINDVDTGVPSI...MALKGTLLLSTLWFKPPFLSD...YTMCEYPNTICNPNLGNQRVELWHWDTGVVSCL
FJ425188_1994     KCTTVSINDVDTGVPSI...MALKGTLLLSTLWFKPPFLSD...YTMCEYPNTICNPNLGNQRVELWHWDTGVVSCL
FJ425189_1994     KCTTVSINDVDTGVPSI...MALKGTLLLSTLWFKPPFLSD...YTMCEYPNTICNPNLGNQRVELWHWDTGVVSCL
FJ425190_1994     KCTTVSINDVDTGVPSI...MALKGTLLLSTLWFKPPFLSD...YTMCEYPNTICNPNLGNQRVELWHWDTGVVSCL
FJ938066_1996     KCTTVSINDVDTGVPSI...MALKGTLLLSTLWFKPPFLSD...YTMCEYPNTICNPNLGNQRVELWHWDTGVVSCL
FJ938063_1996     KCTTVSINDVDTGVPSI...MALKGTLLLSTLWFKPPFLSD...YTMCEYPNTICHSNLGNRRVELWHWDTGVVSCL
NC_003045_1997    KCTTVSINDVDTGVPSI...MALKGTLLLSTLWFKPPFLSD...YTMCEYPNTICNPNLGNQRVELWHWDTGVVSCL
FJ938064_2000     KCTTLSINDVDTGVPSI...MALKGTLLLSTLWFKPPFLSD...YTMCEYPNTICNPNLGNQRVELWHWDTGVVSCL
FJ938065_2000     KCTTLSINDVDTGVPSI...MALKGTLLLSTLWFKPPFLSD...YTMCEYPNTICNPNLGNQRVELWHWDTGVVSCL
LC494164_2006     KCTTVSINDVDTGVPSI...MALKGTLLLSTLWFKPPFLSD...YTMCEYPNTICNPNLGNQRVELWHWDTGVVSCL
LC494162_2006     KCTTVSINDVDTGVPSI...MALKGTLLLSTLWFKPPFLSD...YTMCEYPNTICNPNLGNQRVELWHWDTGVVSCL
LC494163_2006     KCTTVSINDVDTGVPSI...MALKGTLLLSTLWFKPPFLSD...YTMCEYPNTICNPNLGNQRVELWHWDTGVVSCL
LC494160_2007     KCTTVSINDVDTGVPSI...MALKGTLLLSTLWFKPPFLSD...YTMCEYPNTICNPNLGNQRVELWHWDTGVVSCL
LC494161_2007     KCTTVSINDVDTGVPSI...MALKGTLLLSTLWFKPPFLSD...YTMCEYPNTICNPNLGNQRVELWHWDTGVVSCL
LC494172_2007     KCTTVSINDVDTGVPSI...MALKGTLLLSTLWFKPPFLSD...YTMCEYPNTICNPNLGNQRVELWHWDTGVVSCL
LC494173_2008     KCTTVSINDVDTGVPSI...MALKGTLLLSTLWFKPPFLSD...YTMCEYPNTICNPNLGNQRVELWHWDTGVVSCL
LC494174_2008     KCTTVSINDVDTGVPSI...MALKGTLLLSTLWFKPPFLSD...YTMCEYPNTICNPNLGNQRVELWHWDTGVVSCL
LC494177_2008     KCTTVSINDVDTGVPSI...MALKGTLLLSTLWFKPPFLSD...YTMCEYPNTICNPNLGNQRVELWHWDTGVVSCL
LC494165_2008     KCTTVSINDVDTGVPSI...MALKGTLLLSTLWFKPPFLSD...YTMCEYPNTICNPNLGNQRVELWHWDTGVVSCL
LC494166_2008     KCTTVSINDADTGVPSI...MALKGTLLLSTLWFKPPFLSD...YTMCEYPNTICNPNLGNRRVELWHWDTGVVSCL
LC494167_2008     KCTTVSINDADTGVPSI...MALKGTLLLSTLWFKPPFLSD...YTMCEYPNTICNPNLGNRRVELWHWDTGVVSCL
LC494168_2009     KCTTVSINDVDTGVPSI...MALKGTLLLSTLWFKPPFLSD...YTMCEYPNTICNPNLGNQRVELWHWDTGVVSCL
LC494169_2009     KCTTVSINDADTGVPSI...MALKGTLLLSTLWFKPPFLSD...YTMCEYPNTICNPNLGNQRVELWHWDTGVVSCL
LC494170_2010     KCTTVSINDVDTGVPFI...MALKGTLLLSTLWFKPPFLSD...YTMCEYPNTICNPNLGNQRVELWHWDTGVVSCL
LC494171_2010     KCTTVSINDVDTGVPFI...MALKGTLLLSTLWFKPPFLSD...YTMCEYPNTICNPNLGNRRVELWHWDTGVVSCL
LC494129_2010     KCTTVSINDVDTGVPSI...MALKGTLLLSTLWFKPPFLSD...YTMCEYPNTICNPNLGNQRVELWHWDTGVVSCL
LC494130_2010     KCTTVSINDADTGVPSI...MALKGTLLLSTLWFKPPFLSD...YTMCEYPNTICNPNLGNQRVELWHWDTGVVSCL
LC494131_2011     KCTTVSINDVDTGVPSI...MALKGTLLLSTLWFKPPFLSD...YTMCEYPNTICNPNLGNQRVELWHWDTGVVSCL
LC494132_2011     KCTTVSINNVDTGVPSI...MALKGTLLLSTLWFKPPFLSD...YTMCEYPNTICNPNLGNQRVELWHWDTGVVSCL
LC494133_2011     KCTTVSINDADTGVPSI...MALKGTLLLSTLWFKPPFLSD...YTMCEYPNTICNPNLGNQRVELWHWDTGVVSCL
LC494135_2012     KCTTVSINDVDTGVPSI...MALKGTLLLSTLWFKPPFLSD...YTMCEYPNTICNPNLGNQRLELWHWDTGVVSCL
LC494136_2012     KCTTVSINDVDTGVPSI...MALKGTLLLSTLWFKPPFLSD...YTMCEYPNTICNPNLGNQRLELWHWDTGVVSCL
LC494127_2012     KCTTVSINDVDTGVPSI...MALKGTLLLSTLWFKPPFLSD...YTMCEYPNTICNPNLGNQRVELWHWDTGVVSCL
LC494128_2012     KCTTVSINDVDTGVPSI...MALKGTLLLSTLWFKPPFLSD...YTMCEYPNTICNPNLGNQRVELWHWDTGVVSCL
LC494134_2012     KCTTVSINDVDTGVPSI...MALKGTLLLSTLWFKPPFLSD...YTMCEYPNTICNPNLGNQRVELWHWDTGVVSCL
LC494137_2013     KCTTVSINDVDTGVPSI...MALKGTLLLSTLWFKPPFLSD...YTMCEYPNTICNPNLGNQRVELWHWDTGVVSCL
MG757138_2014     KCTTVSINDVDTGVPSI...MALKGTLLLSTLWFKPPFLSD...YTMCEYPNTICNPNLGNQRVELWHWDTGVVSCL
MG757139_2014     KCTTVSINDVDTGVPSI...MALKGTLLLSTLWFKPPFLSD...YTMCEYPNTICNPNLGNQRVELWHWDTGVVSCL
MG757140_2014     KCTTVSINDVDTGVPSI...MALKGTLLLSTLWFKPPFLSD...YTMCEYPNTICNPNLGNQRVELWHWDTGVVSCL
MG757141_2014     KCTTVSINDVDTGVPSI...MALKGTLLLSTLWFKPPFLSD...YTMCEYPNTICNPNLGNQRVELWHWDTGVVSCL
MG757142_2014     KCTTVSINDVDTGVPSI...MALKGTLLLSTLWFKPPFLSD...YTMCEYPNTICNPNLGNQRVELWHWDTGVVSCL
KX982264_2014     KCTTVSINDVDTGVPSI...MALKGTLLLSTLWFKPPFLSD...YTMCEYPNTICNPNLGNQRVELWHWDTGVVSCL
LC494154_2014     KCTTVSINDVDTGVPSI...MALKGTLLLSTLWFKPPFLSD...YTMCEYPNTICNPNLGNQRLELWHWDTGVVSCL
LC494155_2014     KCTTVSINDVDTGVPSI...MALKGTLLLSTLWFKPPFLSD...YTMCEYPNTICNPNLGNQRLELWHWDTGVVSCL
LC494156_2014     KCTTVSINDVDTGVPSI...MALKGTLLLSTLWFKPPFLSD...YTMCEYPNTICNPNLGNQRVELWHWDTGVVSCL
LC494138_2015     KCTTLSINDVDTGVPSI...MALKGTLLLSTLWFKPPFLSD...YTMCEYPNTICNPNLGNQRVELWHWDTGVVSCL
KU886219_2015     KCTTVSINDVDTGVPSI...MALKGTLLLSTLWFKPPFLSD...YTMCEYPNTICHPTLGNQRVELWHWDTGVVSCL
LC494139_2015     KCTTVSINDVDTGVPSI...MALKGTLLLSTLWFKPPFLSD...YTMCEYPNTICNPNLGNQRVELWHWDTGVVSCL
LC494157_2015     KCTTVSINDVDTGVPSI...MALKGTLLLSTLWFKPPFLSD...YTMCEYPNTICNPNLGNQRVELWHWDTGVVSCL
LC494140_2015     KCTTLSINDVDTGVPSI...MALKGTLLLSTLWFKPPFLSD...YTMCEYPNTICNPNLGNQRVELWHWDTGVVSCL
LC494175_2016     KCTTVSINDVDTGVPSI...MALKGTLLLSTLWFKPPFLSD...YTMCEYPNTICNPNLGNQRVELWHWDTGVVSCL
LC494176_2016     KCTTVSINDVDTGVPSI...MALKGTLLLSTLWFKPPFLSD...YTMCEYPNTICNPNLGNQRVELWHWDTGVVSCL
LC494126_2016     KCTTVSINDVDTGVPSI...MALKGTLLLSTLWFKPPFLSD...YTMCEYPNTICNPNLGNQRVELWHWDTGVVSCL
LC494141_2016     KCTTVSINDVDTGVPSI...MALKGTLLLSTLWFKPPFLSD...YTMCEYPNTICNPNLGNQRVELWHWDTGVVSCL
LC494158_2016     KCTTVSINDVDTGVPSI...MALKGTLLLSTLWFKPPFLSD...YTMCEYPNTICNPNLGNQRVELWHWDTGVVSCL
LC494146_2016     KCTTVSINDVDTGVPSI...MALKGTLLLSTLWFKPPFLSD...YTMCEYPNTICNPNLGNQRVELWHWDTGVVSCL
MH043955_2016     KCTTVSINDVDTGVPSI...MALKGTLLLSTLWFKPPFLSD...YTMCEYPNTICNSNLGNQRVELWHWDTGVVSCL
LC494147_2016     KCTTVSINDVDTGVPSI...MALKGTLLLSTLWFKPPFLSD...YTMCEYPNTICNPNLGNQRVELWHWDTGVVSCL
LC494142_2016     KCTTVSINDVDTGVPSI...MALKGTLLLSTLWFKPPFLSD...YTMCEYPNTICNPNLGNQRVELWHWDTGVVSCL
LC494143_2016     KCTTVSINDVDTGVPSI...MALKGTLLLSTLWFKPPFLSD...YTMCEYPNTICNPNLGNRRVELWHWDTGVVSCL
```

```
LC494144_2016    KCTTVSINDVDTGVPSI...MALKGTLLLSTLWFKPPFLSD...YTMCEYPNTICNPNLGNRRVELWHWDTGVVSCL
LC494145_2016    KCTTVSINDVDTGVPSI...MALKGTLLLSTLWFKPPFLSD...YTMCEYPNTICNPNLGNQRVELWHWDTGVVSCL
LC494148_2016    KCTTVSINDVDTGVPSI...MALKGTLLLSTLWFKPPFLSD...YTMCEYPNTICNPNLGNQRVELWHWDTGVVSCL
LC494149_2016    KCTTVSINDVDTGVPSI...MALKGTLLLSTLWFKPPFLSD...YTMCEYPNTICNPNLGNQRVELWHWDTGVVSCL
LC494178_2016    KCTTVSINDVDTGVPSI...MALKGTLLLSTLWFKPPFLSD...YTMCEYPNTICNPNLGNQRVELWHWDTGVVSCL
LC494179_2016    KCTTVSINDVDTGVPSI...MALKGTLLLSTLWFKPPFLSD...YTMCEYPNTICNPNLGNQRVELWHWDTGVVSCL
LC494180_2016    KCTTVSINDVDTGVPSI...MALKGTLLLSTLWFKPPFLSD...YTMCEYPNTICNPNLGNQRVELWHWDTGVVSCL
LC494181_2016    KCTTVSINDVDTGVPSI...MALKGTLLLSTLWFKPPFLSD...YTMCEYPNTICNPNLGNQRVELWHWDTGVVSCL
LC494182_2016    KCTTVSINDVDTGVPSI...MALKGTLLLSTLWFKPPFLSD...YTMCEYPNTICNPNLGNQRVELWHWDTGVVSCL
LC494150_2017    KCTTVSINDVDTGVPSI...MALKGTLLLSTLWFKPPFLSD...YTMCEYPNTICNPNLGNQRVELWHWDTGVVSCL
LC494151_2017    KCTTVSINDVDTGVPSI...MALKGTLLLSTLWFKPPFLSD...YTMCEYPNTICNPNLGNQRVELWHWDTGVVSCL
LC494152_2017    KCTTVSINDVDTGVPSI...MALKGTLLLSTLWFKPPFLSD...YTMCEYPNTICNPNLGNQRVELWHWDTGVVSCL
LC494153_2017    KCTTVSINDVDTGVPSI...MALKGTLLLSTLWFKPPFLSD...YTMCEYPNTICNPNLGNQRVELWHWDTGVVSCL
MG518518_2017    KCTTVSINDVDTGVPTI...MALKGTLLLSTLWFKPPFLSD...YTMCEYPNTICNPNLGNQRVELWHWDTGVVSCL
MH043952_2017    KCTTVSINDVDTGVPSI...MALKGTLLLSTLWFKPPFLSD...YTMCEYPNTICNSNLGNQRVELWHWDTGVVSCL
MH043953_2017    KCTTVSINDVDTGAPSI...MALKGTLLLSTLWFKPPFLSD...YTMCEYPHTICHPNLGNKRVELWHWDTGVVSCL
MH043954_2017    KCTTVSINDVDTGVPSI...MALKGTLLLSTLWFKPPFLSD...YTMCEYPNTICNSNLGNQRVELWHWDTGVVSCL
LC494159_2017    KCTTVSINDVDTGVPSI...MALKGTLLLSTLWFKPPFLSD...YTMCEYPNTICNPNLGNQRVELWHWDTGVVSCL
LC494183_2017    KCTTVSINDVDTGVPSI...MALKGTLLLSTLWFKPPFLSD...YTMCEYPNTICNPNLGNQRVELWHWDTGVVSCL
LC494184_2017    KCTTVSINDVDTGVPSI...MALKGTLLLSTLWFKPPFLSD...YTMCEYPNTICNPNLGNQRVELWHWDTGVVSCL
LC494185_2017    KCTTVSINDVDTGVPSI...MALKGTLLLSTLWFKPPFLSD...YTMCEYPNTICNPNLGNQRVELWHWDTGVVSCL
LC494186_2017    KCTTVSINDVDTGVPSI...MALKGTLLLSTLWFKPPFLSD...YTMCEYPNTICNPNLGNQRVELWHWDTGVVSCL
LC494187_2017    KCTTVSINDVDTGVPSI...MALKGTLLLSTLWFKPPFLSD...YTMCEYPNTICNPNLGNQRVELWHWDTGVVSCL
LC494188_2017    KCTTVSINDVDTGVPSI...MALKGTLLLSTLWFKPPFLSD...YTMCEYPNTICNPNLGNQRVELWHWDTGVVSCL
LC494189_2017    KCTTVSINDVDTGVPSI...MALKGTLLLSTLWFKPPFLSD...YTMCEYPNTICNPNLGNQRVELWHWDTGVVSCL
LC494190_2017    KCTTVSINDVDTGVPSI...MALKGTLLLSTLWFKPPFLSD...YTMCEYPNTICNPNLGNKRVELWHWDTGVVSCL
LC494191_2017    KCTTVSINDVDTGVPSI...MALKGTLLLSTLWFKPPFLSD...YTMCEYPNTICNPNLGNQRVELWHWDTGVVSCL
LC494192_2017    KCTTVSINDVDTGVPSI...MALKGTLLLSTLWFKPPFLSD...YTMCEYPNTICNPNLGNRRVELWHWDTGVVSCL
                 ****:***:.***.* *   *********************   *******:***:..***:*:*************
```

Supplementary Figure 5. Atomic details of the interaction between the RDB domain of HCoV-229E spike protein (class I in black, I-II in light green, IV in light blue, V in salmon and VI in yellow) and hANPEP (in violet) at the binding interface. Salt bridges and H-bonds are represented by dashed blue lines, whereas hydrophobic contacts are indicated as dashed yellow lines. N atoms are colored in blue, O atoms in red. Positively selected sites are underlined. RBDs-hANPEP conserved interaction pattern, mainly involving the 314-320 segment of RBD loop 1 and the 287-292 portion of hANPEP domain II, is shown in panel A. Side chains not involved in the reported interactions have been omitted for clarity, as well as the C317-C320 disulfide bond. Other relevant structural differences among the various RBDs and details on their interactions at the binding interface, involving loop1, loop2 and loop3, are represented in panels B, C and D, respectively. In particular, in loop1 the motif GGG (313-315) of class I is converted in GVG in classes I-II and IV and GPG in classes V and VI. The increased steric hinderance should favor dispersive interactions with the receptor. Additionally, the presence of proline in place of glycine/valine imposes a slight distortion of loop1 that should strengthen the interactions with hANPEP. In general, the different conformation of the entire loop 1 as found in classes I-II, IV, V and VI with respect to class I, strongly alters the orientation of the 316 residue (lysine or arginine) side chain, allowing the formation of a salt bridge with hANPEP D288 that is not observed in class I RBD. For what concerns loop2, the change of its conformation passing from class I RBD to the other variants stabilizes the salt bridge formed between D315 of hANPEP and site 359 (which is arginine for all classes). As for loop3, in class I RBD S407 forms a long-range polar interaction with K292 of hANPEP while K408 interacts with the carbonyl backbone of E291. Both interactions are lost in classes I-II and IV RBDs. Here, however, there are two leucine residues at sites 404 and 408, which could play a role in forming and/or optimizing hydrophobic interactions (with L318, for instance). Strong polar interactions are restored in classes V and VI, where we find a lysine at 408 position, intercepting E291 backbone in hANPEP, and a histidine at site 407, which could form an additional polar interaction with the spatially close D315 of hANPEP. All these observations indicate that specific mutations (especially in loop 3) and changes in the three loops conformation should progressively favor the formation of the hRBD and hANPEP complex going from class I to VI.

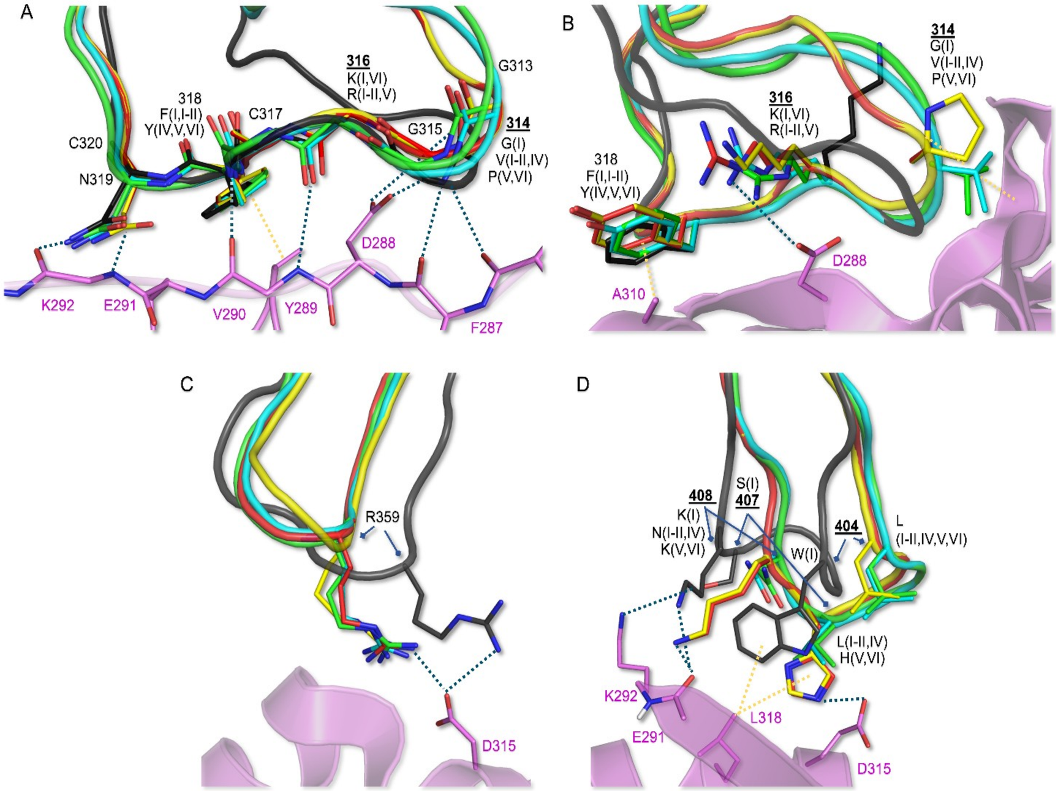

Supplementary Figure 6. Epitopes mapped on the spike protein of different HCoV-OC43 strains, representing different time-frames (from left to right: reference strain, 1967-1990, 1991-2000, 2002-2010, 2011-2019). The proteins are in different shades of gray, whereas the epitopes are in red. The 9-O-acetylated sialic is shown with green spheres.

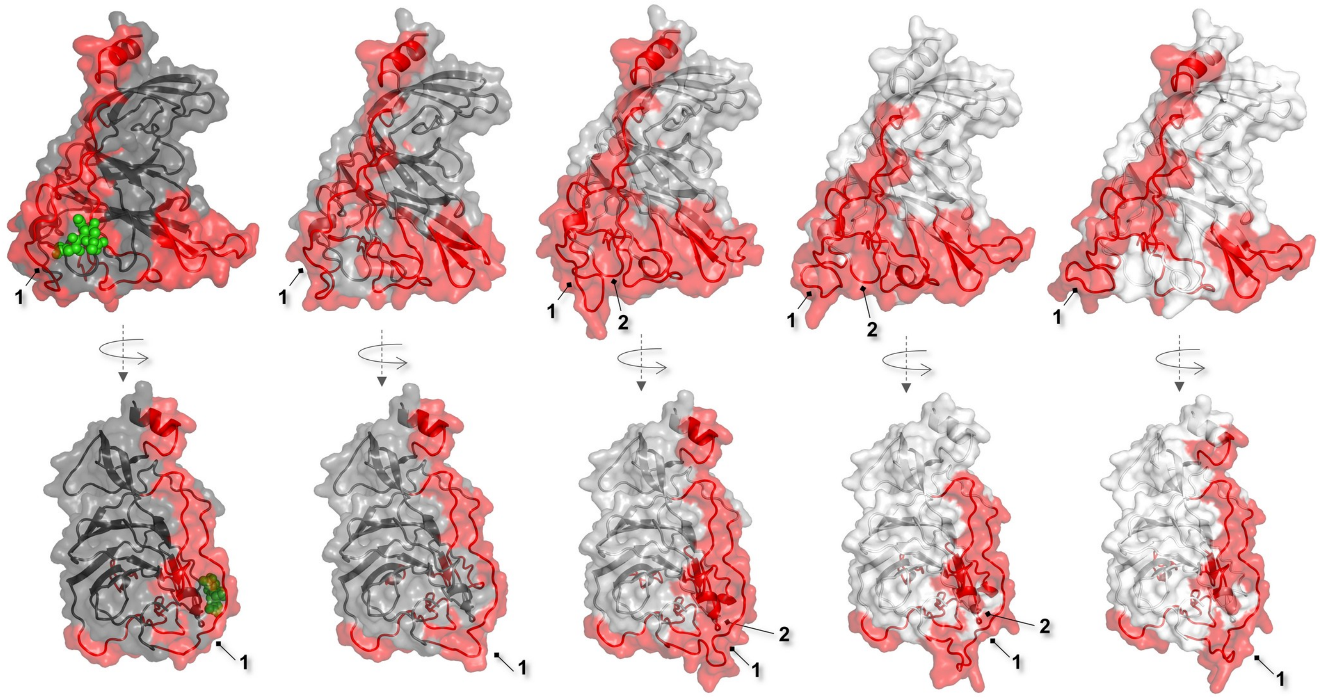

Supplement: veab061_Supp [file veab061_supp.zip › Supplementary_material.pdf]
